# Hemicyanine-Based Wash-Free Near-Infrared Fluorescent Probe for Imaging Amyloid-β Aggregates in Alzheimer’s Disease

**DOI:** 10.3390/molecules31203624

**Published:** 2026-10-11

**Authors:** Xueqi Zhao, Rundong Liang, Yao Li, Jiawei Yan, Jing Zhang, Zheng Su, Yang Liu, Huali Yang, Maosheng Cheng

**Affiliations:** Key Laboratory of Structure-Based Drug Design & Discovery of Ministry of Education, School of Pharmaceutical Engineering, Shenyang Pharmaceutical University, Shenyang 110016, China961050002@syphu.edu.cn (Z.S.); y.liu@syphu.edu.cn (Y.L.)

**Keywords:** Alzheimer’s disease, NIRF Probe, wash-free, log *P*, fluorescence imaging, Aβ aggregates

## Abstract

As a key biomarker of Alzheimer’s disease (AD), amyloid-β (Aβ) is frequently employed for the diagnosis of AD. Near-infrared fluorescence (NIRF) imaging is a non-invasive, low-cost route for visualizing Aβ in vivo. However, most existing Aβ NIRF probes require tedious washing steps, show modest fluorescence turn-on (<50-fold), and stain normal tissue non-specifically. In this work, by fine-tuning the amphiphilicity of a hemicyanine scaffold to place its log *P* within the appropriate range, we identified P3 as a wash-free NIRF probe for Aβ aggregates. P3 bound Aβ aggregates with high affinity. In vivo, P3 distinguished AD mice from wild-type mice with a 4.8-fold signal ratio, together with adequate brain uptake (1.5 ± 0.1 ID/g at 20 min) and rapid clearance. Unlike Thioflavin T, which indiscriminately labels Aβ aggregates and normal neurons, P3 specifically labeled Aβ aggregates with negligible background, which enabled wash-free imaging with a signal-to-noise ratio of 80 ± 4. This work provides a wash-free, high-affinity NIRF probe for Aβ imaging.

## 1. Introduction

Alzheimer’s disease (AD), a neurodegenerative disorder, imposes heavy burdens on families and society [1,2,3]. Although the U.S. Food and Drug Administration (FDA) has approved a number of drugs for the treatment of AD [4], delayed diagnosis means patients often present with overt symptoms; therefore, these drugs merely provide symptomatic relief. A major hindrance to the efficient deployment of AD therapies in the general population is the lack of clinically validated diagnostic methods that use objective, disease-associated biomarkers [5]. Therefore, achieving “early detection and early intervention” to enable drug therapy within a more effective therapeutic time window is of critical importance. The amyloid cascade hypothesis is the prevailing hypothesis for AD pathogenesis, and the aggregation and deposition of amyloid-β (Aβ) is considered a key pathological hallmark for early AD diagnosis [6]. Abnormal Aβ accumulation can occur up to 20 years before clinical symptom onset [7,8,9]. Direct visualization of Aβ aggregates enables the diagnosis of AD prior to the manifestation of clinical symptoms, thereby greatly enhancing the potential for early diagnosis and intervention.

For the detection of Aβ aggregates in clinical practice, magnetic resonance imaging (MRI), positron emission tomography (PET), single-photon emission tomography (SPET), cerebrospinal fluid (CSF) analysis, and blood tests have been developed [10,11,12,13]. Nevertheless, these methods are limited by invasiveness, radiation risk, high cost, and the requirement for advanced instrumentation. In contrast, near-infrared fluorescence (NIRF) probes, offering the advantages of low cost, non-invasiveness, and high safety, have gradually become a major research focus in the field of biological imaging [14,15,16,17,18,19,20,21,22].

**Figure 1:**
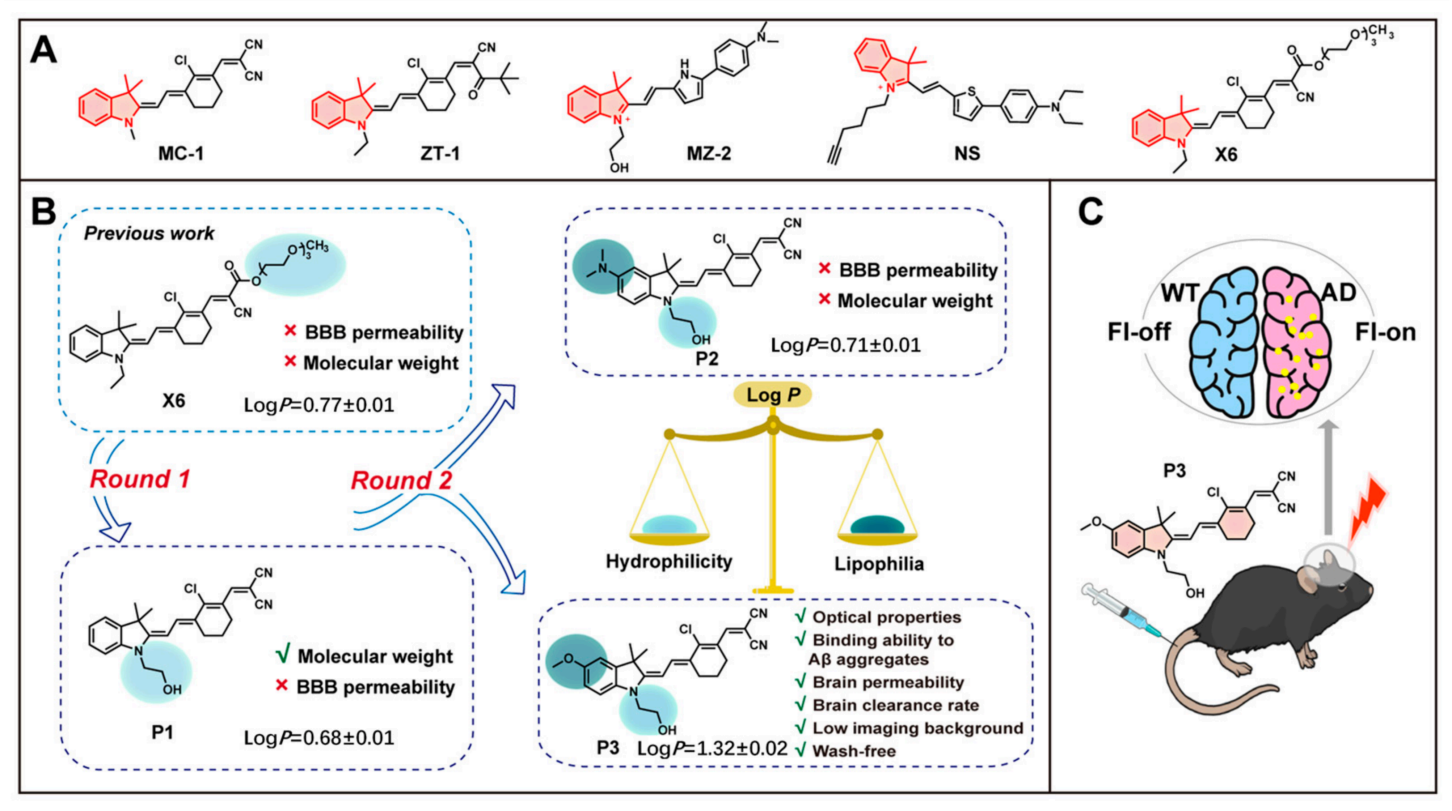
(**A**) Chemical structures of hemicyanine probes targeting Aβ aggregates. (**B**) Schematic illustration of the two rounds of structural modifications of the probe. (**C**) The distinction between WT mice and AD mice by P3.

In recent years, the donor–π–acceptor (D–π–A) scaffold has been widely employed in the development of NIRF probes. The D–π–A skeleton enables sensitive microenvironmental responses through intramolecular charge transfer-controlled fluorescence on/off switching [23,24,25,26,27,28,29,30,31,32,33,34,35,36,37,38,39,40]. Owing to the favorable optical properties, good biocompatibility, and low toxicity, hemicyanine dyes represent ideal core candidates for D–π–A type probes. However, most reported hemicyanine-based Aβ probes still suffer from three key limitations (Figure 1A, Table S1). First, their fluorescence enhancement upon Aβ binding is modest (<50-fold). Second, their Stokes shifts are small (<100 nm), which elevates imaging background [41,42,43,44,45]. Third, and most importantly, nearly all of them require tedious washing steps before imaging because they stain normal tissue non-specifically; this washing requirement severely limits their use in real-time or in situ Aβ detection. The development of a wash-free Aβ NIRF probe with a high signal-to-noise ratio (SNR) therefore remains an unmet need. Amphiphilicity has recently emerged as a practical handle for constructing wash-free Aβ probes (Table S3). Zhang et al. combined a cyano group with amphipathic modulation to produce NIR-I Aβ probes that bind two distinct sites of Aβ fibrils and give ultra-high signal-to-noise ratios even without washing [46]. More recently, Li et al. tuned the amino end groups of a styrene pyridinium scaffold and balanced its hydrophilicity and hydrophobicity, obtaining wash-free probes with improved plaque labeling [47]. Together with related amphiphilic probe designs, these studies establish amphiphilicity control as a viable route to wash-free Aβ imaging. However, they were developed on specific non-hemicyanine scaffolds, and their amphiphilicity was adjusted largely in an empirical manner rather than being directed towards a defined log *P* window. For hemicyanine-based Aβ probes, wash-free behavior therefore remains to be systematically addressed.

The log *P* of a probe is a key determinant of its brain permeability, in vivo clearance, and nonspecific binding, all of which collectively contribute to the imaging background [46,47,48]. Here, we report a strategy that fine-tunes log *P* by adjusting probe amphiphilicity through the cooperative use of lipophilic groups and a hydroxyethyl group. Starting from our previously reported hemicyanine probe X6, which showed satisfactory in vitro Aβ binding but limited in vivo responsiveness owing to its low log *P* [45], two rounds of structural modification afforded P1, P2, and P3 (Figure 1B). P3, which carries a methoxy group, achieved the appropriate log *P* of 1.32 ± 0.02. Benefiting from this favorable log *P*, P3 functions as a wash-free NIRF probe that specifically labels Aβ aggregates with negligible background and high SNR. This work thus provides a practical tool for Aβ imaging.

## 2. Results and Discussion

### 2.1. Probe Design and Structural Optimization

**Scheme 1:**
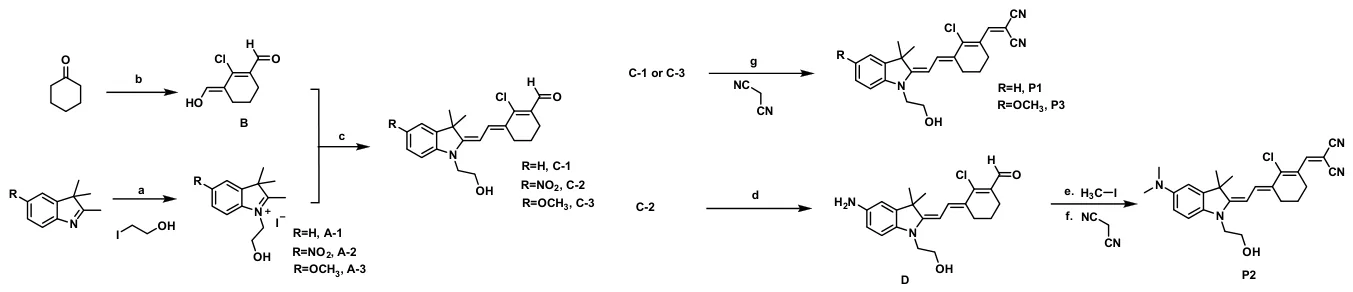
The synthetic routes for probes. Reagents and conditions: (**a**) toluene, 110 °C, 10 h; (**b**) dry DCM, dry DMF, POCl_3_, 80 °C, 6 h; (**c**) CH_3_COOH, toluene, 110 °C, 3.5 h; (**d**) Fe, NH_4_Cl, EtOH, 80 °C, 2 h; (**e**) acetonitrile, 80 °C, 24 h; (**f**,**g**) piperidine, dry DCM, r.t., 15 min.

All probes employ a hemicyanine scaffold with a D–π–A architecture. P1 was designed from X6 by replacing the polyethylene glycol chain on the indole nitrogen with a hydroxyethyl group and introducing a cyano group. This reduced the molecular weight by approximately one quarter and strengthened the electron-withdrawing ability of the D-π-A system. However, the measured log *P* of P1 was lower than that of X6, indicating that the hydroxyethyl group increased hydrophilicity rather than enhancing brain permeability. Consequently, further structural modifications were performed on P1. In the second round, lipophilic groups were introduced onto the indole ring of P1: an N,N-dimethyl group afforded P2, and a methoxy group afforded P3. These lipophilic groups were intended to cooperate with the hydroxyethyl group to raise log *P* and balance brain permeability with clearance, while the strong electron donors (N,N-dimethyl and methoxy) enhanced the electron-donating capacity of the D-π-A system and further red-shifted the emission (Figure 1B). The synthetic routes of probes P1, P2, and P3 are shown in Scheme 1. The synthesis of these probes involved N-alkylation, Vilsmeier–Haack–Arnold reaction, condensation reaction, reduction reaction, and Knoevenagel condensation. Detailed synthetic procedures are described in Section 3.1 (Synthesis of Probe).

### 2.2. Density Functional Theory (DFT) Calculations of Probe

**Figure 2:**
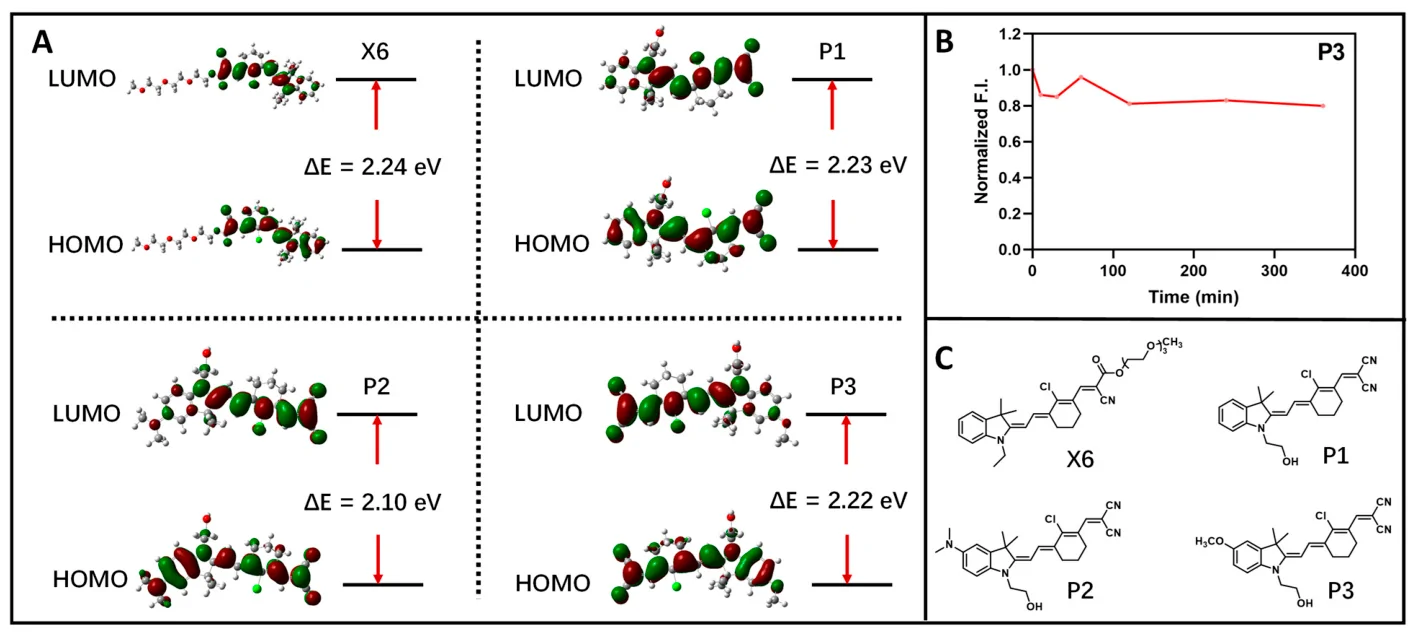
(**A**) DFT calculation of the probe. (**B**) The fluorescence intensity of P3 was tested after different illumination times (0 min, 10 min, 30 min, 60 min, 120 min, 240 min and 360 min). (**C**) Structure of the probe.

Density functional theory (DFT) calculations on Probes (X6, P1, P2 and P3) were carried out using the Gaussian program (Figure 2A), and their highest occupied molecular orbital (HOMO) and lowest unoccupied molecular orbital (LUMO) energies were computed to obtain the HOMO–LUMO energy gap (Δ*E_g_*). A narrower energy gap corresponds to a more red-shifted emission. The calculated Δ*E_g_* values were 2.24 eV for X6, 2.23 eV for P1, 2.10 eV for P2, and 2.22 eV for P3. The decrease from X6 to P1 theoretically reflects the red-shift caused by the stronger electron-withdrawing malononitrile group in the D–π–A system, whereas the further decreases in P2 and P3 relative to P1 indicate additional red-shifts induced by the introduction of strong electron-donating groups.

### 2.3. Optical Properties of Probe

The optical properties of probes were evaluated, as shown in Table 1, Figure 2B, Figures S18 and S19 (Supporting Information). In phosphate-buffered saline (PBS), the optimal emission maxima were 780 nm for P1, 819 nm for P2, and 785 nm for P3. Relative to X6, P1 exhibited red-shifted wavelengths; P2 and P3 showed further red-shifts compared to P1, confirming the theoretical predictions from DFT calculations. Their large Stokes shifts (164 nm for P2, 135 nm for P3) indicate low imaging background. Subsequently, the light stability of probes was evaluated (Figure 2B and Figure S19), and the results confirmed that all probes possess strong photostability, which provides a reliable basis for stable imaging performance. Absolute fluorescence quantum yield (Φ) reflects the conversion of absorbed light to fluorescence, determines signal intensity, and is a key probe-performance metric. Since dichloromethane (DCM) can mimic the hydrophobic pocket microenvironment of Aβ aggregates, the Φ values of the probes were measured in DCM (Table 1 and Figure S20). P3 exhibited a higher Φ (2.34 ± 0.10%) than X6 (1.03%), suggesting far superior fluorescence emission efficiency in DCM. This indicates that upon Aβ binding, P3 suppresses non-radiative decay more effectively, converting more absorbed energy into fluorescence and thus is well-suited for imaging.

### 2.4. Log P Test of Probe

The log *P* values were then determined (Table 1, Figure S21). P1 (0.68 ± 0.01) showed a lower log *P* than X6 (0.77 ± 0.01), confirming the predominantly hydrophilic nature of the hydroxyethyl group. The increased log *P* of P2 (0.71 ± 0.01) and P3 (1.32 ± 0.02) relative to P1 experimentally validated the strategy of combining lipophilic groups with the hydroxyethyl group for fine-tuning log *P*.

### 2.5. Affinity to Aβ Aggregates of Probe In Vitro

**Figure 3:**
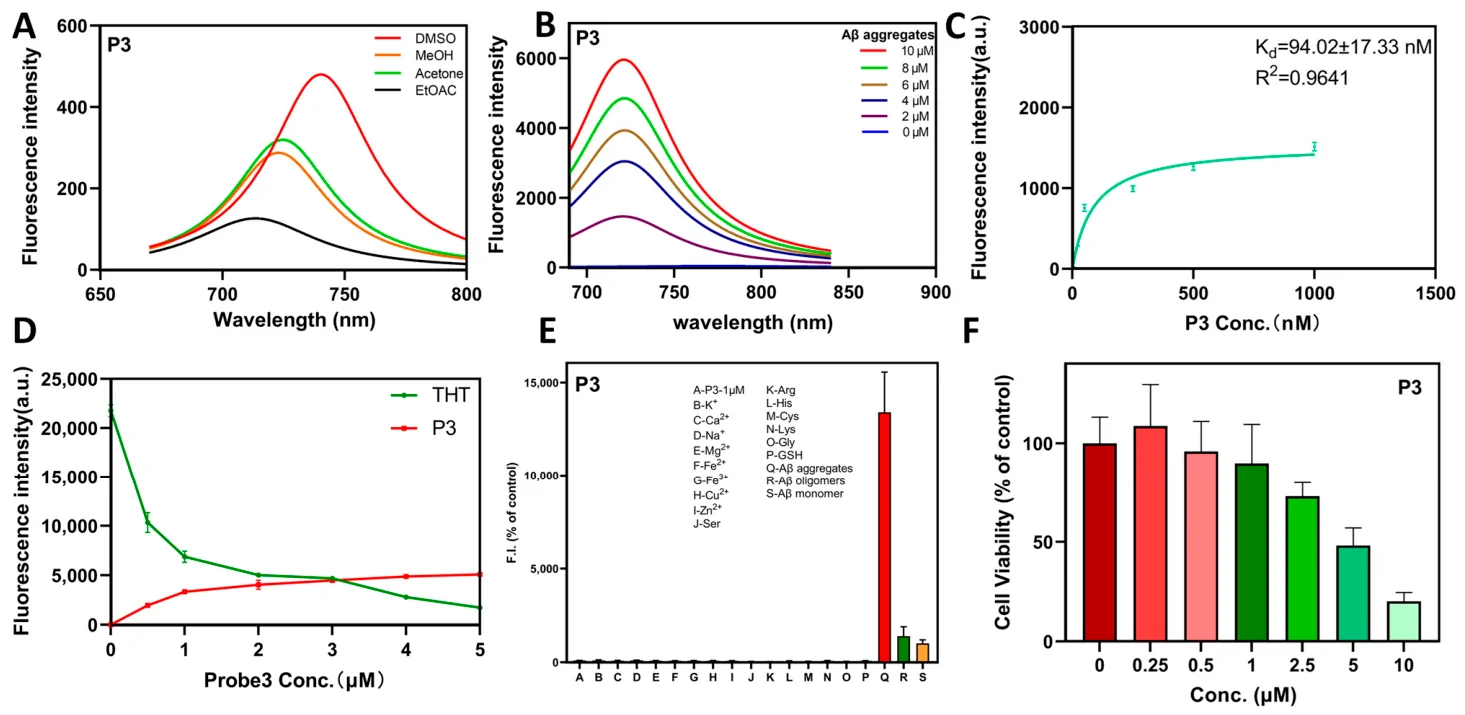
(**A**) The fluorescence spectra of P3 in different solvents (DMSO, MeOH, Acetone, EtOAC). (**B**) The fluorescence spectra of 1 μM P3 in different concentrations of Aβ aggregates (0, 2, 4, 6, 8, 10 μM). (**C**) The fluorescence titration of P3 with A β aggregates (3 μM) using a saturation binding model. (**D**) P3 (0, 0.5, 1, 2, 3, 4, 5 μM) replaced ThT in the ThT @A β (5 μM/10 μM) aggregate complex. λ_P3_ = 720 nm, λ_ThT_ = 482 nm. (**E**) The fluorescence response of P3 (1 μM) to various potential interferers (10 μM): K^+^, Ca^2+^, Na^+^, Mg^+^, Fe^2+^, Fe^3+^, Cu^2+^, Zn^2+^, Ser, Arg, His, Cys, Lys, Gly, GSH, Aβ aggregates, Aβ oligomers, Aβ monomer. All the experiments mentioned above were conducted in phosphate-buffered saline (pH 7.4); λex = 650 nm. (**F**) The cell viability of SH-SY5Y cells after incubation with different concentrations (0, 0.25, 0.5, 1, 2.5, 5, 10 μM) of P3. Error bars: mean ± standard deviation (SD) (*n* = 3).

Polarity sensitivity assays (Figure 3A and Figure S18) showed that all probes exhibited blue-shifted emission with decreasing solvent polarity, indicating their sensitivity to the polarity of the surrounding environment. We therefore reasonably speculated that, upon successful entry into the hydrophobic cavity of Aβ aggregates, the emission maxima of the probes would be blue-shifted relative to those in PBS. Subsequently, the in vitro binding affinity of probes toward Aβ aggregates was evaluated. The emission spectra of probes exhibited varying degrees of blue shift (P1 = 780 nm→ 715 nm; P2 = 819 nm→ 780 nm; P3 = 785 nm→ 720 nm). These results were consistent with the polarity sensitivity tests, confirming that the probes successfully entered the hydrophobic cavities of Aβ aggregates. The fold increase is defined as the ratio of the fluorescence intensity of the probe (1 μM) after binding to Aβ aggregates (10 μM) to that before binding. Remarkably, P3 displayed a 131.4 ± 7.0-fold fluorescence enhancement, which was vastly superior to the 40.0 ± 0.6-fold enhancement of X6 (Table 1, Figure S22). Accordingly, P3 was selected as the optimal candidate for subsequent investigations. Next, the concentration-dependent binding of P3 to Aβ aggregates was evaluated (Figure 3B and Figure S23). The results revealed a concentration-dependent increase in fluorescence intensity, indicating that P3 can respond to Aβ aggregates at different concentrations with high sensitivity, thus providing a foundation for the quantitative analysis of Aβ aggregates.

The dissociation constant (K_d_) of P3 was evaluated quantitatively through fluorescence titration using a saturation binding model (Figure 3C and Figure S24). The K_d_ value of P3 was calculated as 94.02 ± 17.33 nM, demonstrating favorable binding affinity for Aβ aggregates. To further assess the binding affinity of P3 to Aβ aggregates, Thioflavin T (ThT) was utilized to perform the binding competition experiment (Figure 3D). P3 at different concentrations was gradually added to a mixture of ThT binding with Aβ (ThT @Aβ), and the fluorescence intensity was measured. The results showed that with increasing concentrations of P3, the fluorescence intensity of ThT at 482 nm progressively decreased, while that of P3 at 785 nm gradually increased. This finding demonstrates the competitive binding of P3 and ThT to Aβ aggregates. Specificity is one of the important parameters to evaluate the performance of the probes. Thus, the binding selectivity of P3 was assessed. P3 was incubated with various metal ions, amino acids, Aβ aggregates, Aβ monomer, and Aβ oligomer, followed by fluorescence intensity measurements (Figure 3E and Figure S25). The results showed that among the competitive factors that may have an impact on the selected test, P3 showed high selectivity for Aβ aggregates. Biocompatibility was evaluated using SH-SY5Y neuronal cells with the CCK-8 assay. Cells were incubated with P3 (0–10 μM), and viability remained high at concentrations up to 2.5 μM (Figure 3F), indicating favorable biosafety for subsequent animal studies.

**Figure 4:**
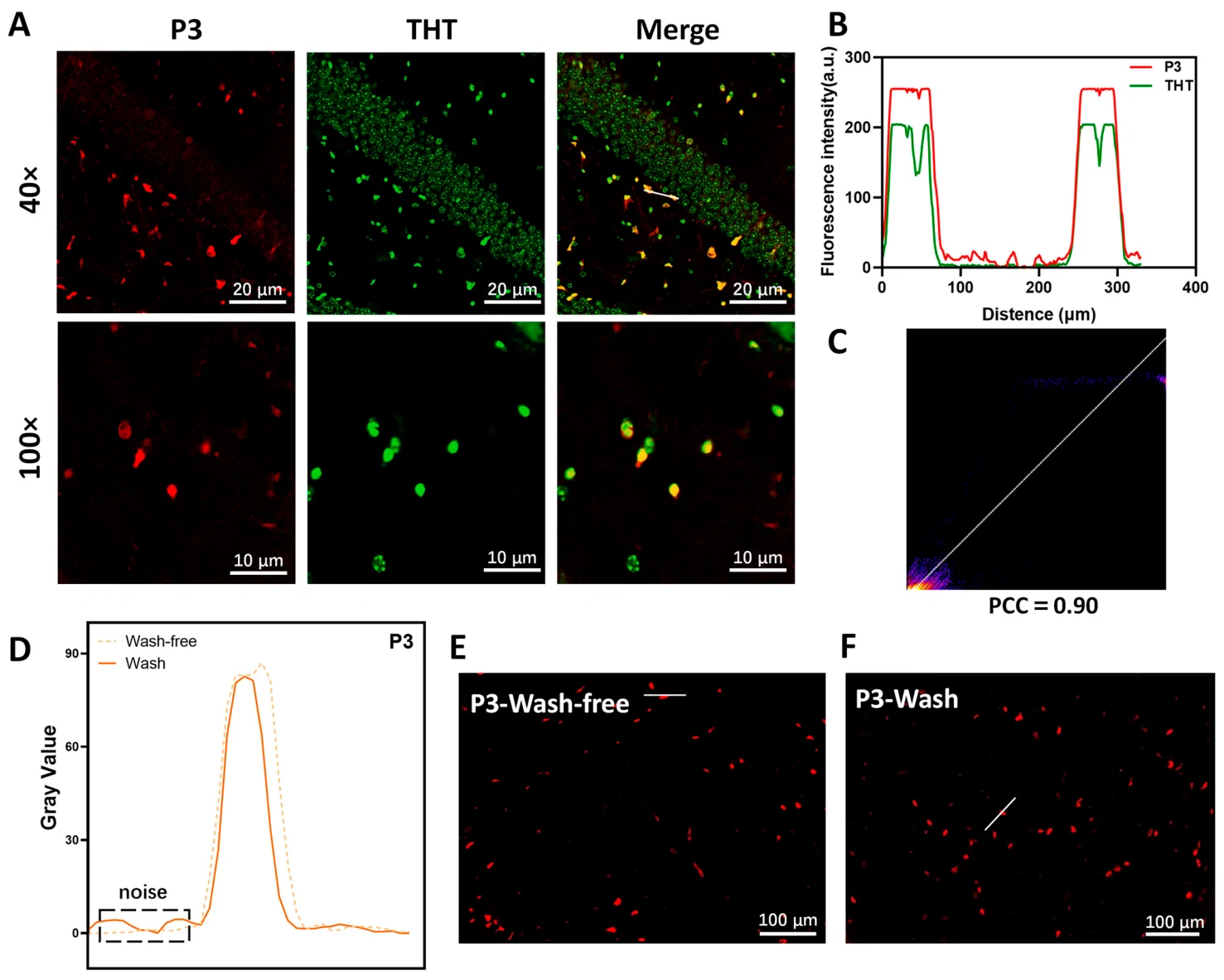
(**A**) In vitro co-localization fluorescence images staining with ThT and P3 of Aβ aggregates in AD mouse brain slices. (**B**) The intensity profiles of linear regions of interest that intersect the brain sections. (**C**) Pearson’s correlation coefficient map of P3. (**D**) The SNR of P3 at the white line area on the images of panels (**E**,**F**). In vitro fluorescence images staining with P3 of Aβ aggregates in AD mouse brain slices. (**E**) Staining without washing, (**F**) washing after staining.

To further investigate the labeling performance of P3 at the tissue level, co-staining experiments were performed on AD brain slices (APP/PS1, 10 months old) using commercial ThT as a reference standard. P3 and ThT were co-incubated on the same brain slice (Figure 4A). Confocal imaging showed strong fluorescence signals in both the green channel (ThT) and the red channel (P3). The merged image confirmed successful co-localization of P3 and ThT on Aβ aggregates, demonstrating that P3 enables high-fidelity imaging of Aβ aggregates in brain tissue. Notably, P3 showed richer Aβ aggregate feedback than ThT. Compared to ThT, P3 could amplify the fidelity signal (Figure 4B). The merged image revealed colocalization between P3 and ThT (Pearson’s correlation coefficient r = 0.90), indicating that the P3 demonstrates comparable precision to the commercial probe in locating Aβ aggregates. Notably, in the green channel for ThT, it was observed that ThT not only labeled Aβ aggregates but also indiscriminately stained normal neuronal cell bodies (Figure 4A). This represents a critical drawback of ThT for Aβ labeling: its poor specificity generates inherent background noise, severely compromising detection accuracy. In contrast, P3 did not label normal cells, and its red channel showed extremely weak background, allowing for clear imaging observation. Given the excellent Aβ labeling performance and negligible imaging background of P3, its wash-free imaging capability was further evaluated. Images of AD brain slices stained with P3 with and without washing were compared (Figure 4E,F). Both washed and unwashed slices displayed strong Aβ aggregate labeling signals. Under the washing condition, P3 achieved a high SNR of 89 ± 6. Notably, even under the wash-free condition, P3 maintained a high SNR of 80 ± 4 with negligible background interference (Figure 4D). This demonstrates that P3 can effectively label Aβ aggregates without the need for washing. This high SNR is primarily attributed to the appropriate hydrophilicity of P3, which minimizes nonspecific binding in non-pathological areas, as well as its high affinity for Aβ aggregates. Such high-performance wash-free probes have rarely been reported, and the wash-free staining assay may serve as an important criterion for evaluating in vitro labeling performance.

### 2.6. Affinity to Aβ Aggregates of the Probe In Vivo

**Figure 5:**
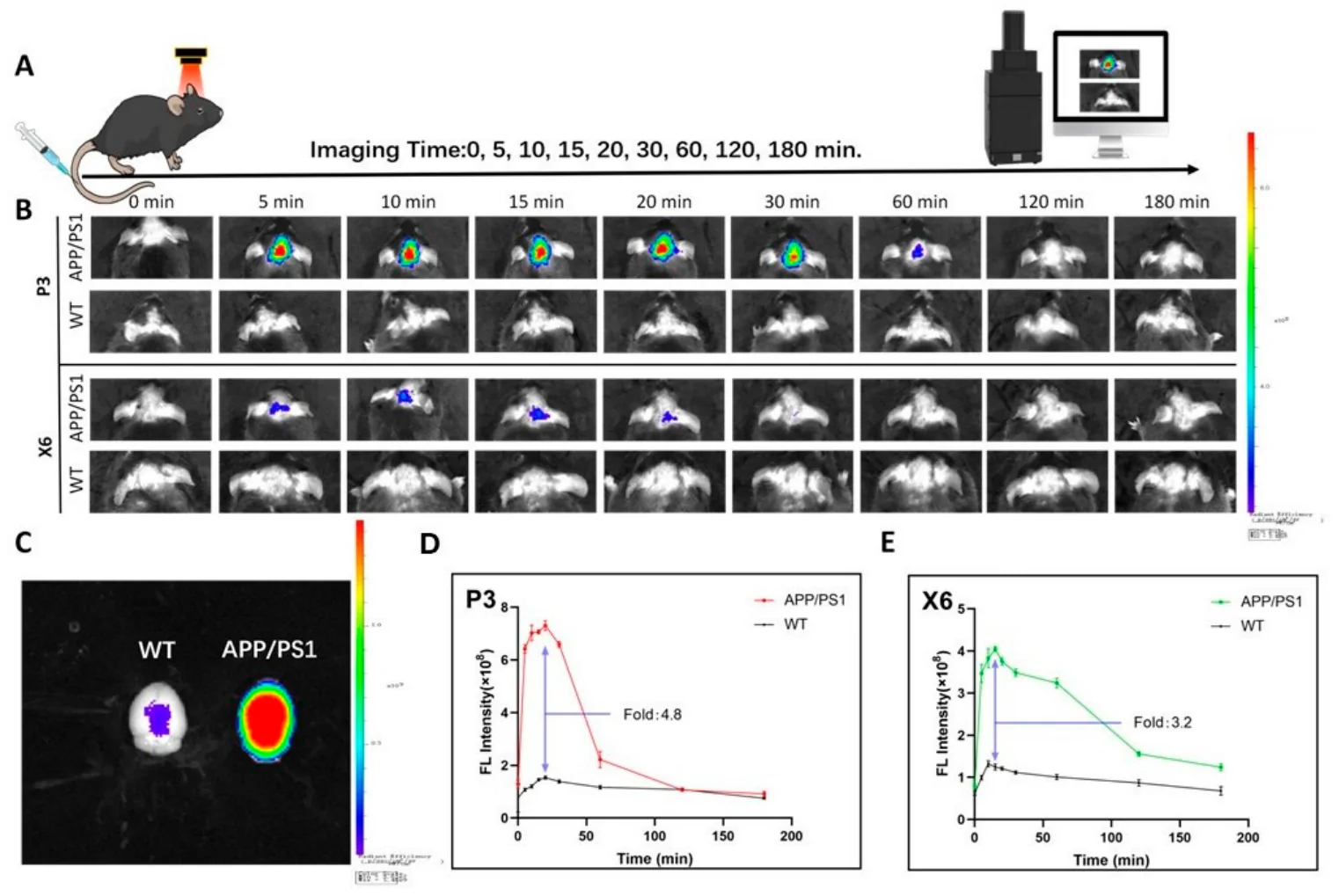
(**A**) The time flow chart of in vivo imaging. (**B**) In vivo NIRF imaging of AD mice and WT mice at representative time points before and after intravenous injection of P3 and X6, respectively. P3λex = 650 nm, λem = 720 nm. X6λex = 610 nm, λem = 708 nm. (**C**) Brain tissue imaging after intravenous injection of P3. (**D**,**E**) Quantitative analysis of the fluorescence signal was performed to obtain the brain dynamics curves of P3 and X6 at the selected time points. Error bars: mean ± standard deviation (SD) (*n* = 3).

In vivo NIRF brain imaging was performed on 10-month-old APP/PS1 (AD) and age-matched WT mice. After intravenous injection of P3 or X6 (4 mg/kg), brain fluorescence was monitored at various post-injection (Figure 5A,B). Clear signals from P3 appeared as early as 5 min, with higher intensity in AD than in WT mice. P3 peaked at 20 min, while X6 peaked at 15 min; under identical settings, P3 showed markedly stronger fluorescence than X6. Quantitative analysis at peak intensity revealed a 4.8-fold signal ratio between AD and WT mice for P3, versus 3.2-fold for X6, demonstrating that P3 effectively crosses the blood–brain barrier and labels Aβ aggregates in vivo with superior imaging capability (Figure 5D,E). In addition, AD mice and WT mice were intravenously injected with P3 (4 mg/kg). Twenty minutes after injection, the mice were sacrificed, and the brains were harvested for imaging (Figure 5C). P3 exhibited stronger fluorescence labeling in AD mice, further confirming its brain-targeting ability and specific labeling capacity for Aβ aggregates.

**Figure 6:**
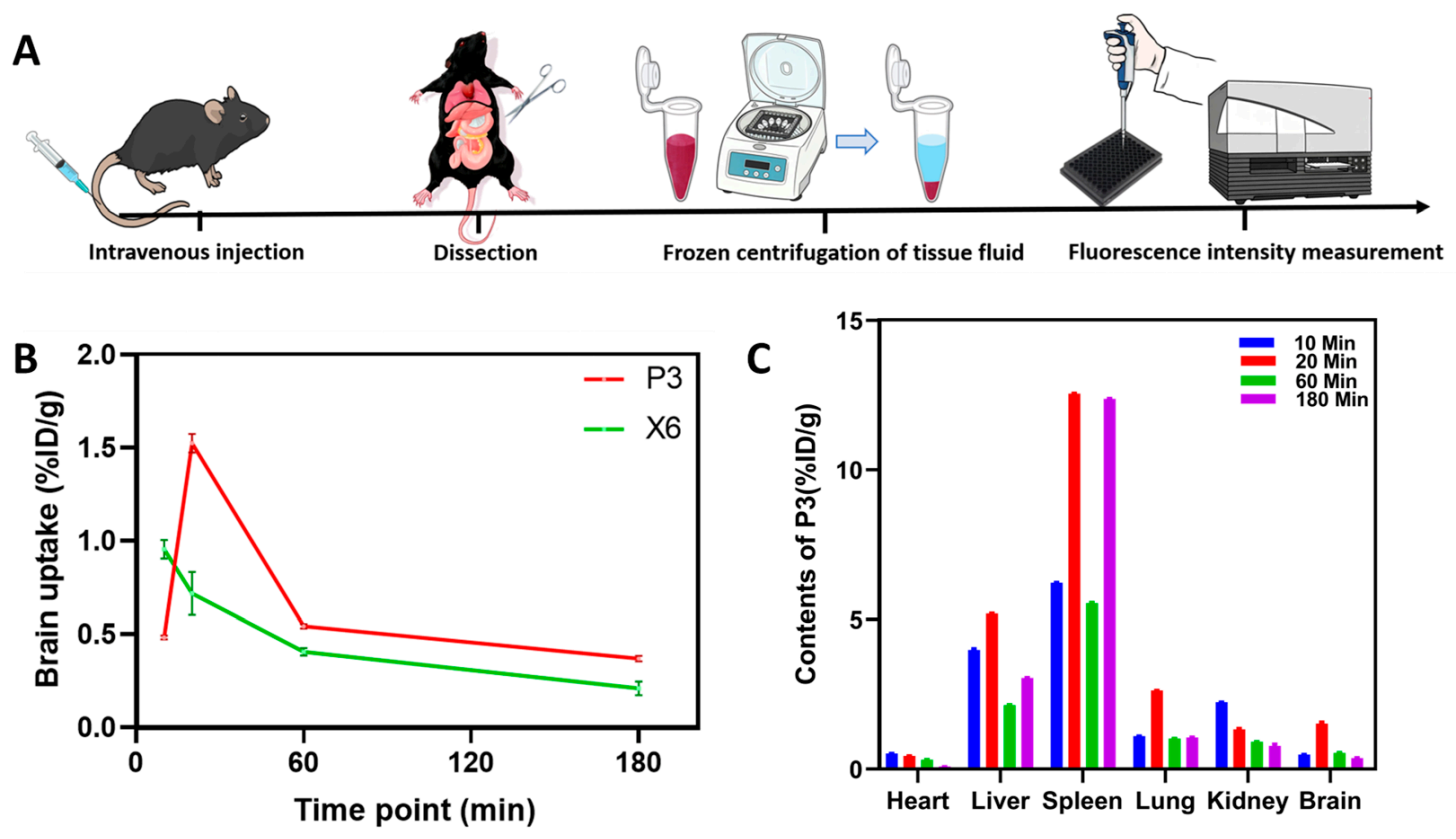
(**A**) The experimental flow chart of brain uptake, brain clearance rate and tissue distribution. (**B**) The brain exposure of P3 and X6 was measured at 10, 20, 60, and 180 min after injection into C57 mice, respectively. (**C**) The tissue distribution of P3 after injection into C57 mice (4 mg/kg). Error bars: mean ± standard deviation (SD) (*n* = 3).

A suitable log *P* balances lipophilicity and hydrophilicity: sufficient lipophilicity ensures adequate brain uptake, while appropriate hydrophilicity enables rapid brain clearance and minimizes background from nonspecific binding. P3, with a log *P* of 1.32 ± 0.02, has shown high Aβ binding affinity and low background in vitro and in vivo. To further assess its advantages, brain uptake and clearance were quantified (Figure 6B and Figure S26). P3 brain uptake and clearance were quantified using a standard curve generated from C57 mouse brain homogenates. After injection of P3 (4 mg/kg), C57 mice were sacrificed at 10, 20, 60, and 180 min; brain supernatants were analyzed to determine P3 concentration and calculate % injected dose per gram (ID/g). P3 peaked at 20 min with 1.5 ± 0.1% ID/g, exceeding X6 (0.9% ± 0.1 ID/g at 10 min), demonstrating superior brain uptake and imaging capability (Figure 6B). P3 exhibited rapid brain clearance (Brain_20min_/Brain_180min_ = 4.16 vs. 3.44 for X6). Tissue distribution analysis (Figure 6C and Figure S27) showed predominant splenic accumulation. P3 levels in heart, lungs, kidneys, and brain gradually declined, whereas liver and spleen levels rebounded at 3 h (especially spleen), likely due to mononuclear phagocyte system-mediated redistribution and enterohepatic circulation.

**Figure 7:**
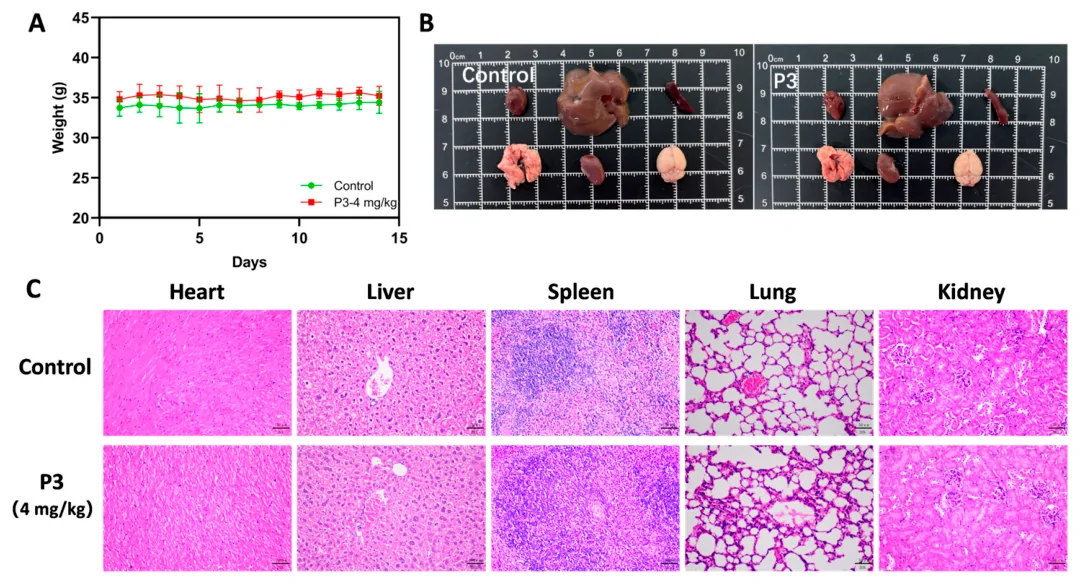
(**A**) The body weight changes in C57 mice in the control group and P3 group within 14 days. Error bars: mean ± standard deviation (SD) (*n* = 3). (**B**) Visceral appearance of C57 mice in the control group and P3 group after anatomy. (**C**) HE staining sections of visceral tissues of mice in the control group and P3 group.

To evaluate the in vivo toxicity of P3, C57 mice were intravenously injected with P3 or saline (4 mg/kg). No significant body weight changes were observed over 14 days (Figure 7A). On day 15, gross and histological (H&E) examinations of major organs revealed no abnormalities in either group (Figure 7B,C), confirming P3 was well tolerated at the dose tested.

### 2.7. Molecular Docking

**Figure 8:**
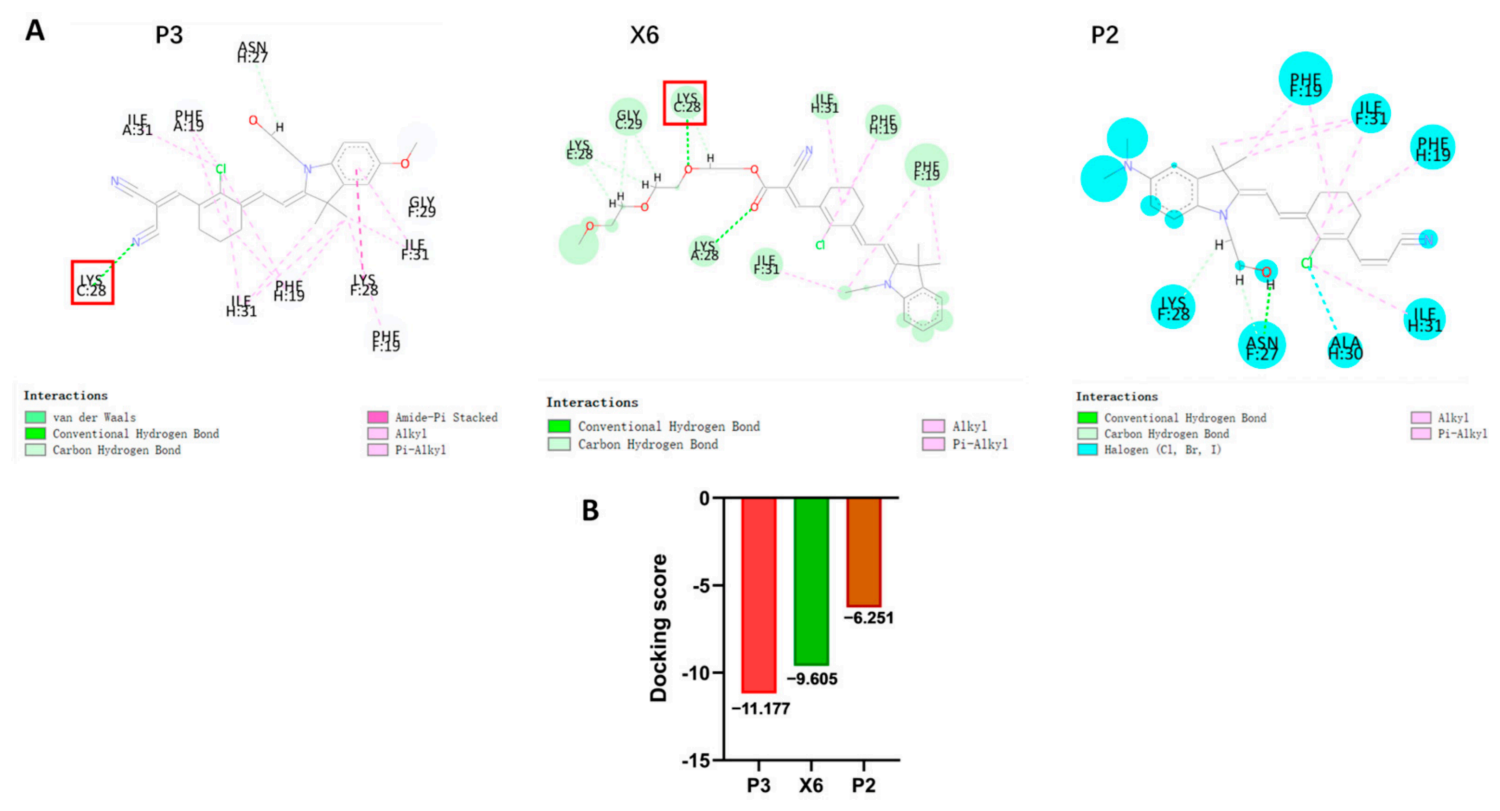
(**A**) The docking mode of X6, P2 and P3 with Aβ_1–42_ aggregates (PDB: 5OQV). (**B**) The docking score of X6, P2 and P3 with Aβ_1–42_ aggregates.

**Figure 9:**
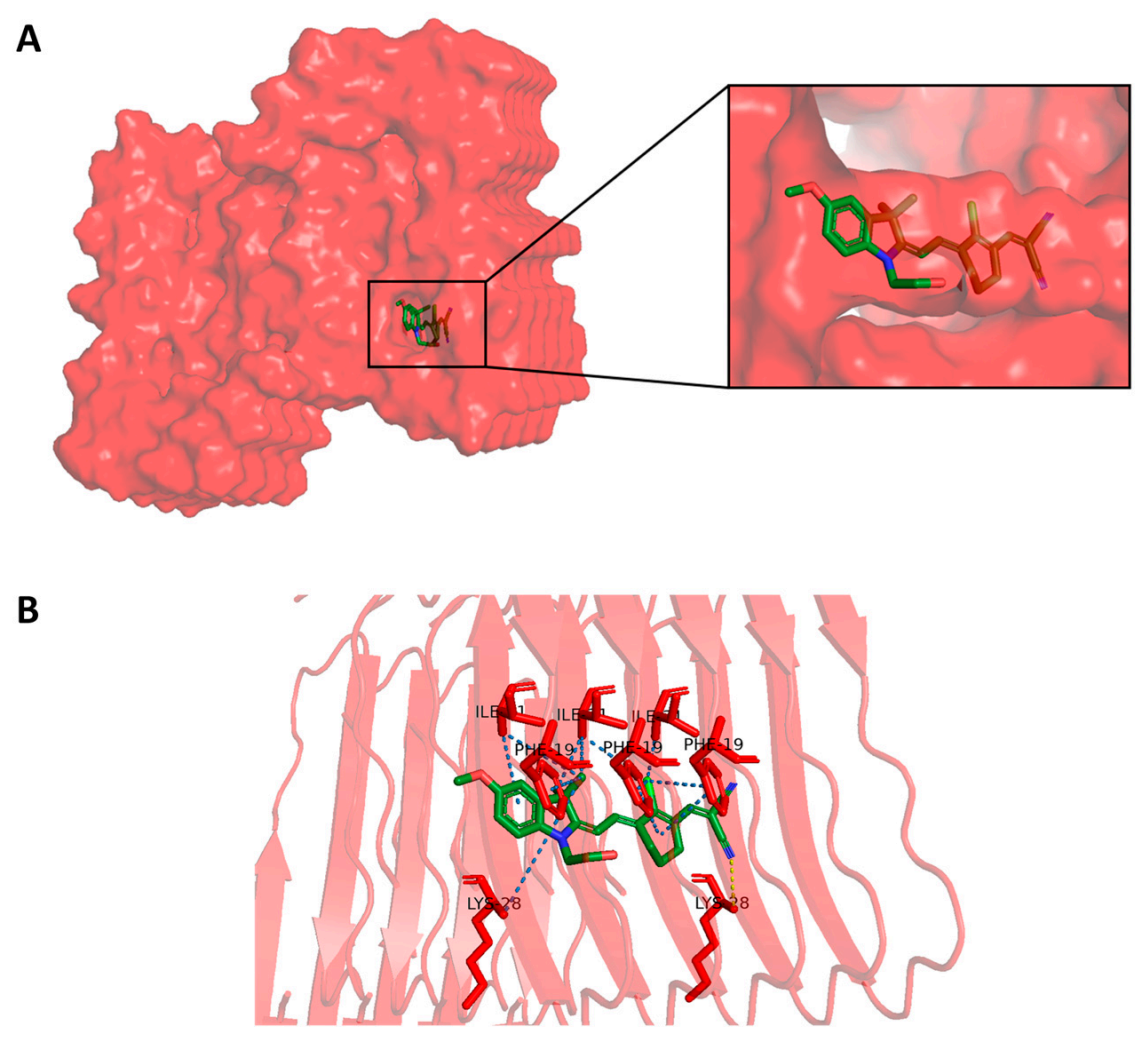
(**A**) The docking diagram and docking amplification diagram of the optimal compound P3 and Aβ_1–42_ aggregates. (**B**) The docking diagram and interaction mode of P3 and Aβ_1–42_ aggregates.

Molecular docking of X6, P2, and P3 (Figure 8 and Figure 9) showed that P3 exhibited stronger binding than X6, while P2 had the weakest affinity, consistent with in vitro and in vivo binding assays. Interaction mode analysis (Figure 8A) was further performed to guide future structural optimization. Compared with X6, P3 retained the hydrogen bond with Lys28 of chain C, gained amide-π stacking with Lys28 of chain F, and formed significantly more hydrophobic interactions. P2 formed a hydrogen bond with Asn of chain F and a halogen bond with Ala of chain H but had fewer hydrophobic contacts, likely due to steric hindrance from the *N*,*N*-dimethyl group. These findings highlight the essential hydrogen bond with Lys28 (chain C) and the critical role of hydrophobic interactions; maximizing such interactions is an effective strategy for enhancing probe–Aβ binding.

## 3. Materials and Methods

### 3.1. Synthesis of Probe

The raw materials and solvents used were analytically pure and purchased from suppliers for the experiment, requiring no further purification. The reaction was monitored by using a thin-layer chromatography silica gel plate (Yantai Jiangyou Silica Gel Development Co., Ltd., Yantai, China). The silica gel used in column chromatography was produced by Qingdao Ocean Chemical Co., Ltd., Qingdao, China, using an NMR spectrometer ((AVANCE) III HD 600 or 400 MHz, Bruker BioSpin, Fällanden, Switzerland). ^1^H and ^13^C NMR spectra were measured. The ^1^H and ^13^C NMR spectra were recorded in CDCl_3_ and DMSO-d_6_ solutions at room temperature. From tetramethylsilane to the chemical shift, (δ) was recorded at a low ppm field, and the coupling constant (J) was reported in Hertz (Hz). The multiplicity was defined as “s” (single peak). d (double peak), t (triple peak), or m (multiple peak). High-resolution quality spectrum was measured by a Waters Xevo G2-XS Tof.

#### 3.1.1. Synthesis of 1-(2-Hydroxyethyl)-2,3,3-trimethyl-3H-indol-1-ium (**A-1**)

2-iodoethanol (1.5 g, 8.79 mmol) and 2,3,3-trimethyl-3H-indole (1 g, 6.28 mmol) were refluxed in acetonitrile for 24 h. The reaction liquid was cooled to room temperature, and n-hexane was added dropwise, filtered and dried to obtain 1.1 g of purple solid, namely intermediate A-1, with a yield of 85.27%. ^1^H NMR (400 MHz, DMSO-d_6_) δ 7.99–7.90 (m, 1H), 7.88–7.81 (m, 1H), 7.62 (dd, *J* = 5.8, 3.1 Hz, 2H), 4.60 (t, *J* = 5.1 Hz, 2H), 3.88 (t, *J* = 5.1 Hz, 2H), 2.82 (s, 3H), 1.55 (s, 6H). ESI-MS(m/z): 205.1 [M+H]^+^.

#### 3.1.2. Synthesis of 1-(2-Hydroxyethyl)-2,3,3-trimethyl-5-nitro-3H-indol-1-ium (**A-2**)

2-iodoethanol (12.64 g, 73.50 mmol) and 2,3,3-trimethyl-5-nitro-3H-indole (10 g, 48.96 mmol) were refluxed in acetonitrile for 12 h. The reaction liquid was cooled to room temperature, and n-hexane was added dropwise, filtered and dried to obtain 4 g of yellow-green solid, namely intermediate A-2, with a yield of 21.73%. ^1^H NMR (400 MHz, DMSO-d_6_) δ 8.06 (dd, *J* = 8.8, 2.4 Hz, 1H), 7.99 (d, *J* = 2.4 Hz, 1H), 7.02 (d, *J* = 8.7 Hz, 1H), 3.91–3.78 (m, 2H), 3.60 (qd, *J* = 9.2, 8.2, 4.0 Hz, 2H), 1.38 (s, 3H), 1.33 (d, *J* = 5.0 Hz, 6H). ESI-MS(m/z): 250.1 [M+H]^+^.

#### 3.1.3. Synthesis of 1-(2-Hydroxyethyl)-5-methoxy-2,3,3-trimethyl-3H-indol-1-ium (**A-3**)

2-iodoethanol (10.18 g, 59.20 mmol) and 5-methoxy-2,3,3-trimethyl-3H-indole (8 g, 42.27 mmol) were refluxed in acetonitrile for 12 h. The reaction liquid was cooled to room temperature, and n-hexane was added dropwise, filtered and dried to obtain brown solid 5.8 g, namely intermediate A-3, with a yield of 38.01%. ^1^H NMR (400 MHz, DMSO-d_6_) δ 7.85 (d, *J* = 8.9 Hz, 1H), 7.49 (d, *J* = 2.5 Hz, 1H), 7.14 (dd, *J* = 8.8, 2.6 Hz, 1H), 5.25 (s, 1H), 4.55 (t, *J* = 5.1 Hz, 2H), 3.97 − 3.73 (m, 5H), 2.75 (s, 3H), 1.53 (s, 6H). ESI-MS(m/z): 235.1 [M+H]^+^.

#### 3.1.4. Synthesis of 2-Chloro-3-[(E)-hydroxymethylidene]cyclohex-1-ene-1-carbaldehyde (**B**)

Phosphorus oxychloride (7.5 mL, 80.46 mmol) was added dropwise to a mixed solution containing 9 mL anhydrous DCM and 9 mL anhydrous DMF under nitrogen protection and ice bath conditions. After stirring for 30 min, cyclohexanone (2.25 mL, 21.85 mmol) was added and refluxed at 80 °C for 6 h. After cooling to room temperature, the reaction solution was poured into ice water and filtered to obtain a solid 2.64 g, namely intermediate B, with a yield of 70%. ^1^H NMR (600 MHz, Chloroform-*d*) δ 10.44 (s, 1H), 2.72 − 2.67 (m, 2H), 2.61 (t, *J* = 6.0 Hz, 2H), 2.07 (p, *J* = 6.2 Hz, 2H). ESI-MS(m/z): 173.1 [M+H]^+^.

#### 3.1.5. Synthesis of 3-[(1E)-2-[(2E)-1-(2-Hydroxyethyl)-3,3-dimethyl-2,3-dihydro-1H-indol-2-ylidene]ethylidene]-2-chlorocyclohex-1-ene-1-carbaldehyde (**C-1**)

The intermediate A-1 (0.66 g, 2 mmol) and intermediate B (0.35 mg, 2 mmol) were dissolved in a mixed solution containing 2.5 mL glacial acetic acid and 7.5 mL toluene and heated at 100~120 °C for 3 h, and the solvent was removed. The red solid 162.4 mg was obtained by column chromatography, and the intermediate C-1 was obtained with a yield of 22.74%. ^1^H NMR (400 MHz, Chloroform-d) δ 10.24 (s, 1H), 7.81 (d, *J* = 12.8 Hz, 1H), 7.21 (t, *J* = 7.2 Hz, 2H), 6.95 (t, *J* = 7.4 Hz, 1H), 6.82 (d, *J* = 7.9 Hz, 1H), 5.62 (d, *J* = 12.6 Hz, 1H), 3.96 (d, *J* = 5.1 Hz, 2H), 3.94 − 3.86 (m, 2H), 2.67 − 2.53 (m, 2H), 2.46 (d, *J* = 6.9 Hz, 2H), 1.75 (dd, *J* = 13.0, 6.8 Hz, 2H), 1.67 (s, 6H). ESI-MS(m/z): 358.0 [M+H]^+^.

#### 3.1.6. Synthesis of 3-[(1E)-2-[(2E)-1-(2-Hydroxyethyl)-3,3-dimethyl-5-nitro-2,3-dihydro-1H-indol-2-ylidene]ethylidene]-2-chlorocyclohex-1-ene-1-carbaldehyde (**C-2**)

The intermediate A-2 (1 g, 2.66 mmol) and intermediate B (0.459 g, 2.66 mmol) were dissolved in a mixed solution containing 25 mL of glacial acetic acid and 75 mL of toluene, heated at 100 ~ 120 °C for 3 h. After removing the solvent, the red solid 270 mg was obtained by column chromatography, and the intermediate C-2 was obtained with a yield of 25.24%. ^1^H NMR (400 MHz, Chloroform-d) δ 10.27 (s, 1H), 8.16 (dd, *J* = 8.7, 2.2 Hz, 1H), 8.02 (d, *J* = 2.1 Hz, 1H), 7.71 (d, *J* = 12.4 Hz, 1H), 6.82 (d, *J* = 8.7 Hz, 1H), 5.76 (d, *J* = 12.5 Hz, 1H), 4.00 (t, *J* = 5.0 Hz, 2H), 3.95 (d, *J* = 5.3 Hz, 2H), 2.57 (t, *J* = 6.3 Hz, 2H), 2.46 (t, *J* = 6.0 Hz, 2H), 1.82 − 1.73 (m, 2H), 1.70 (s, 6H). ESI-MS(m/z): 403.0 [M+H]^+^.

#### 3.1.7. Synthesis of 3-[(1E)-2-[(2E)-1-(2-Hydroxyethyl)-5-methoxy-3,3-dimethyl-2,3-dihydro-1H-indol-2-ylidene]ethylidene]-2-chlorocyclohex-1-ene-1-carbaldehyde (**C-3**)

The intermediate A-3 (0.6 g, 1.67 mmol) and intermediate B (0.29 g, 1.67 mmol) were dissolved in a mixed solution containing 10 mL glacial acetic acid and 30 mL toluene and heated at 100 ~ 120 °C for 3 h, and the solvent was removed. The red solid 143 mg was obtained by column chromatography, and the intermediate C-3 was obtained with a yield of 22.12%. ^1^H NMR (400 MHz, DMSO-d_6_) δ 10.05 (d, *J* = 1.1 Hz, 1H), 7.74 (dd, *J* = 12.9, 6.2 Hz, 1H), 7.22 (s, 1H), 7.04 (dd, *J* = 6.6, 2.5 Hz, 1H), 6.92 (d, *J* = 8.6 Hz, 1H), 6.78 (dt, *J* = 8.6, 2.1 Hz, 1H), 5.67 (dd, *J* = 12.9, 6.5 Hz, 1H), 4.42 (t, *J* = 4.8 Hz, 3H), 4.14 (s, 2H), 3.73 (d, *J* = 1.2 Hz, 2H), 2.35 (q, *J* = 6.6 Hz, 2H), 2.18 (t, *J* = 6.1 Hz, 2H), 1.63 (s, 2H), 1.57 (s, 6H). ESI-MS(m/z): 388.0 [M+H]^+^.

#### 3.1.8. Synthesis of 3-[(1E)-2-[(2E)-5-Amino-1-(2-hydroxyethyl)-3,3-dimethyl-2,3-dihydro-1H-indol-2-ylidene]ethylidene]-2-chlorocyclohex-1-ene-1-carbaldehyde (**D**)

The intermediate C-2 (0.45 g, 1.11 mmol) was dissolved in 20 mL ethanol. After adding ammonium chloride aqueous solution, iron powder (0.31 g, 5.53 mmol) was added in batches under stirring, and the temperature was raised to reflux temperature. After 2 h of reaction, it was filtered while hot. The filtrate was adjusted to pH 8~9. After extraction with ethyl acetate, the solvent was removed to obtain the crude product, which was separated by column chromatography to obtain 59.5 mg of purple solid, namely intermediate D, with a yield of 14.41%. ^1^H NMR (400 MHz, Chloroform-d) δ 10.24 (d, *J* = 10.5 Hz, 1H), 7.81 (d, *J* = 13.0 Hz, 1H), 6.62 (dd, *J* = 5.3, 2.9 Hz, 1H), 6.55 (dd, *J* = 8.3, 2.2 Hz, 1H), 5.63 (d, *J* = 12.6 Hz, 1H), 5.53 (d, *J* = 12.8 Hz, 1H), 4.12 (q, *J* = 7.1 Hz, 2H), 3.95 (dd, *J* = 13.8, 5.3 Hz, 2H), 3.86 (d, *J* = 5.6 Hz, 2H), 2.56 (s, 2H), 2.48 (d, *J* = 5.6 Hz, 2H), 1.76 (q, *J* = 6.3 Hz, 2H), 1.64 (s, 6H). ESI-MS(m/z): 373.2 [M+H]^+^.

#### 3.1.9. Synthesis of 2-({3-[(1E)-2-[(2E)-1-(2-Hydroxyethyl)-3,3-dimethyl-2,3-dihydro-1H-indol-2-ylidene]ethylidene]-2-chlorocyclohex-1-enyl}methylidene)propanedinitrile (**P1**)

The intermediate C-1 (0.052 g, 0.15 mmol) and malononitrile (0.017 g, 0.26 mmol) were dissolved in anhydrous DCM, and 0.14 mL piperidine was added as a catalyst. The reaction was carried out at room temperature for 15 min, the solvent was removed, and column chromatography was performed to obtain a blue solid 37 mg. The hemicyanine near-infrared fluorescent probe P1 was obtained in a yield of 60.89%. ^1^H NMR (400 MHz, DMSO-d6) δ 8.01 (d, *J* = 13.5 Hz, 1H), 7.80 (s, 1H), 7.49–7.44 (m, 1H), 7.30 (td, *J* = 7.7, 1.2 Hz, 1H), 7.19 (d, *J* = 7.9 Hz, 1H), 7.10 (t, *J* = 7.4 Hz, 1H), 6.01 (d, *J* = 13.6 Hz, 1H), 4.96 (d, *J* = 6.9 Hz, 1H), 4.09–4.03 (m, 2H), 3.72 (d, *J* = 5.3 Hz, 2H), 2.80 (t, *J* = 6.0 Hz, 2H), 2.57 (t, *J* = 6.1 Hz, 2H), 1.83–1.74 (m, 2H), 1.61 (s, 6H). ^13^C NMR (101 MHz, DMSO) δ 168.54, 150.70, 147.31, 143.33, 139.81, 139.75, 133.38, 128.62, 128.07, 122.95, 122.69, 121.99, 118.09, 116.48, 110.06, 97.31, 59.74, 47.60, 45.83, 27.87, 27.66, 26.89, 25.22, 20.76. HRMS (m/z): 406.1685 [M+H]^+^.

#### 3.1.10. Synthesis of 2-({3-[(1E)-2-[(2E)-5-(Dimethylamino)-1-(2-hydroxyethyl)-3,3-dimethyl-2,3-dihydro-1H-indol-2-ylidene]ethylidene]-2-chlorocyclohex-1-enyl}methylidene)propanedinitrile (**P2**)

The intermediate E (0.15 g, 0.37 mmol) and malononitrile (0.042 g, 0.64 mmol) were dissolved in anhydrous DCM, and 0.14 mL piperidine was added as a catalyst. The reaction was carried out at room temperature for 15 min. The solvent was removed, and the blue solid was separated by column chromatography to obtain 48 mg. The hemicyanine near-infrared fluorescent probe P2 was obtained with a yield of 28.96%. ^1^H NMR (400 MHz, DMSO-d6) δ 8.31 (s, 1H), 7.48 (s, 1H), 7.18 (d, *J* = 8.7 Hz, 1H), 6.97 (d, *J* = 2.4 Hz, 1H), 6.69 (dd, *J* = 8.9, 2.4 Hz, 1H), 6.11 (d, *J* = 14.1 Hz, 1H), 4.99 (t, *J* = 5.7 Hz, 1H), 4.14 (t, *J* = 5.5 Hz, 2H), 3.74 (d, *J* = 5.6 Hz, 2H), 2.94 (s, 6H), 2.78 (t, *J* = 6.1 Hz, 2H), 2.54 (d, *J* = 4.9 Hz, 2H), 1.63 (s, 8H). ^13^C NMR (101 MHz, DMSO) δ 170.23, 162.18, 154.82, 149.27, 146.69, 146.18, 142.18, 141.16, 133.09, 121.55, 120.43, 119.17, 111.80, 106.90, 99.61, 58.61, 48.80, 46.67, 27.84, 26.55, 25.30, 20.51. HRMS (m/z): 449.2105 [M+H]^+^.

#### 3.1.11. Synthesis of 2-({3-[(1E)-2-[(2E)-1-(2-Hydroxyethyl)-5-methoxy-3,3-dimethyl-2,3-dihydro-1H-indol-2-ylidene]ethylidene]-2-chlorocyclohex-1-enyl}methylidene)propanedinitrile (**P3**)

The intermediate C-3 (0.14 g, 0.36 mmol) and malononitrile (0.04 g, 0.61 mmol) were dissolved in anhydrous DCM, and 0.14 mL of piperidine was added as a catalyst. The reaction was carried out at room temperature for 15 min. The solvent was removed, and the blue solid was separated by column chromatography to obtain 28.6 mg. The hemicyanine near-infrared fluorescent probe P3 was obtained with a yield of 18.34%. ^1^H NMR (600 MHz, DMSO-d6) δ 8.31 (s, 1H), 8.03 (d, *J* = 13.8 Hz, 1H), 7.66 (s, 1H), 7.18 (dd, *J* = 5.6, 3.0 Hz, 1H), 6.90 (dd, *J* = 8.7, 2.5 Hz, 1H), 6.03 (d, *J* = 13.8 Hz, 1H), 4.98 (t, *J* = 5.9 Hz, 1H), 4.08 (t, *J* = 5.6 Hz, 2H), 3.78 (s, 3H), 3.75 − 3.70 (m, 2H), 2.78 (t, *J* = 6.1 Hz, 2H), 2.55 (t, *J* = 6.2 Hz, 2H), 1.77 (p, *J* = 6.3 Hz, 2H), 1.62 (s, 6H). ^13^C NMR (151 MHz, DMSO-d6) δ 169.79, 156.82, 149.06, 147.04, 141.81, 140.66, 136.64, 122.18, 121.30, 118.91, 117.25, 113.12, 111.22, 108.93, 98.14, 79.19, 58.05, 55.79, 48.31, 46.25, 27.77, 26.80, 25.26, 20.50. HRMS (m/z): 436.1787 [M+H]^+^.

### 3.2. Excitation and Emission Wavelength Test

The probe was accurately weighed and dissolved in DMSO to prepare a 10 mM DMSO stock solution, which was diluted with PBS buffer to 2.0 μM for later use. In a 96-well plate, 200 μL of the test solution was added, and fluorescence detection was performed using a full-band microplate reader (Tecan Austria GmbH, SPARK, Grödig, Austria). The excitation or emission wavelengths were fixed and scanned continuously to determine the excitation and emission wavelengths of the target probe in PBS.

### 3.3. Polarity Sensitivity Test

The probe was accurately weighed and dissolved in DMSO to prepare a 10 mM DMSO stock solution, which was diluted with different solvents (DMSO, MeOH, Acetone, EtOAC) to 2.0 μM for later use. In 96-well plates, 200 μL of test solution was added, the excitation wavelength was fixed, and the fluorescence spectrum was scanned by a full-band microplate reader (Tecan Austria GmbH, SPARK).

### 3.4. Log P Test

Lipophilicity governs passive membrane permeation, brain clearance, and nonspecific tissue binding, whereas probes with a lower log *P* permeate membranes less readily and strongly lipophilic probes tend to bind non-specifically and to clear slowly. The n-octanol/water partition coefficient (log *P*) is defined as the base-10 logarithm of the ratio of the equilibrium concentrations of a compound in the two mutually saturated phases: log *P* = log_10_(C_n-octanol_/C_water_). Firstly, the standard curve of the probe needs to be prepared. The fluorescence curve of the probe in the water-saturated n-octanol solution with the change in concentration was measured. The standard curve of the probe was obtained by linear fitting of the concentration and fluorescence intensity for quantitative analysis. The specific experimental methods are as follows. The probe was dissolved in n-octanol to prepare a 10 mM n-octanol stock solution, which was diluted with water-saturated n-octanol solution. The final concentrations were 1000 μM, 500 μM, 200 μM, 100 μM, 50 μM, 25 μM, 10 μM, 1 μM. In the 96-well blackboard, 200 μL of each diluent was added to each well. When testing Log *P*, the stock solution of each probe was added to 1.0 mL of n-octanol, and the n-octanol solution was fully vortexed with 1.0 mL of n-octanol-saturated water for 5 min and then centrifuged at 3000 rpm for 5 min to make an n-octanol and water layer. The fluorescence intensity of the upper n-octanol solution was measured and the concentration was obtained from the standard curve. The concentration of probe in water is obtained by subtracting the amount of probe in n-octanol from the total amount of probe added, and then dividing by the volume of the water layer.

### 3.5. Light Stability

The 10 mM DMSO stock solution of the probe was irradiated under a white light lamp (λem > 400 nm). The stock solution was taken at 0 min, 10 min, 30 min, 60 min, 120 min, 240 min and 360 min to prepare a 20 μM probe solution, and its fluorescence intensity was measured.

### 3.6. Cytotoxicity Test

SH-SY5Y cells were cultured in DMEM high glucose containing 10% FBS (Viva Cell Biosciences, Shanghai, China), 100 U/mL penicillin and 100 μg/mL streptomycin at 37 °C in a 5% CO_2_ saturated humidity incubator. The cytotoxicity of SH-SY5Y cells was determined by CCK8 assay, which is based on the reduction of the water-soluble tetrazolium salt WST-8 [2-(2-methoxy-4-nitrophenyl)-3-(4-nitrophenyl)-5-(2,4-disulfophenyl)-2H-tetrazolium, monosodium salt] to a water-soluble formazan by cellular dehydrogenases; the amount of formazan produced is directly proportional to the number of viable cells. SH-SY5Y cells were seeded into 96-well plates at a density of 6000 cells per well and incubated for 24 h. DMEM high-glucose medium was contained in each well at a volume of 100 μL. The original medium was discarded, and different concentrations of test compounds (10, 5, 2.5, 1, 0.5, 0.25, 0 μM) diluted in the medium were added. The cells were re-incubated for 24 h, and the CCK8 solution (Beyotime, Shanghai, China) was added to each well at a volume of 20 μL and incubated for 1–2 h. Finally, the absorbance was detected at a wavelength of 450 nm. Cell viability was calculated as (absorbance value of sample − absorbance value of blank)/(absorbance value of control − absorbance value of blank) × 100%. Data were visualized as bar graphs using GraphPad Prism 8.3.0. The results were expressed as mean ± SD of three independent experiments.

### 3.7. Brain Section Staining Test

The brain tissue of APP/PS1 mice was taken for tissue sectioning (paraffin embedding), and the brain sections were stained with fluorescence in vitro. Before staining, the normal dewaxing process is carried out. The sections were washed with PBS (10 mM, pH = 7.4) 3 times, and then incubated with the probe solution (10 μM) for 20 min at room temperature, followed by washing with PBS buffer3 times. After removing the residual liquid with dust-free paper, the sections were stained with ThT solution (10 μM) for 10 min. Finally, the slices were covered with VectaShield fixed medium, and the fluorescence co-localization imaging test was performed on the slices. Fluorescence co-localization imaging was performed by a confocal laser scanning microscope. In the same field of view, ThT channel and probe channel were collected in turn for image superposition and co-localization analysis. The images were analyzed by ImageJ 1.54p. In the wash or wash-free test, brain tissue sections (paraffin-embedded) of APP/PS1 mice were taken for in vitro wash and wash-free imaging of Aβ plaques. The brain slices were washed three times with PBS (10 mM, pH = 7.4), and then incubated with the probe (10 μM) at room temperature for 20 min. Before sealing, the stained brain slices were washed three times with PBS (10 mM, pH = 7.4) or not. Fluorescence imaging was performed by a confocal laser scanning microscope. ImageJ software was used to analyze the data and images.

### 3.8. Tissue Distribution Test

C57 mice of the same litter and sex with the same strain and age as the experimental group were not injected with the probe as the blank group (male C57 mice, n = 3 per group). All mice were tail-marked for identification and then randomly allocated to groups. The allocation was performed before probe administration, and the person who generated the allocation sequence did not take part in the subsequent measurements. No mice were excluded from the analysis. All animals completed the study and no deaths or technical failures occurred. The number of animals reported in the Results therefore equals the number of animals included at the start of the study. Firstly, prepare the standard curve of the probe in each tissue. Remove the brain, heart, liver, spleen, lung, and kidney. Weigh them with an analytical balance, add PBS buffer (5 mL/g), and then subject them to severe homogenization (65 Hz, 240 s) to obtain tissue homogenates. Take each tissue homogenate, and then add equal volumes of methanol and acetonitrile and vortex, so that protein denaturation and extraction take place. Centrifuge the mixture at 14,400 rpm for 5 min. The supernatant was transferred to the EP tube, and the precipitate was dissolved in an equal volume of PBS buffer, and then equal volumes of methanol and acetonitrile were added according to the above operation and then vortexed and centrifuged. The supernatant was combined in the EP tube, and the precipitate was subjected to the same treatment (a total of 3 times) to obtain the supernatant. The probe mother liquor was diluted with this supernatant and configured into different concentrations of probe diluent to obtain fluorescence intensity. The standard curve of the probe was obtained by linear fitting of probe concentration and fluorescence intensity, which was used for the following quantitative study. The probe was injected into the tail vein of healthy male C57 mice (4 mg/kg). The mice were sacrificed by cervical dislocation at 10 min, 20 min, 60 min and 3 h after injection. The brain, heart, liver, spleen, lung and kidney were removed and weighed. The cut tissue was added to PBS buffer (5 mL/g) and then homogenized vigorously (65 Hz, 240 s) to obtain the homogenate of each tissue. The homogenate of each tissue was taken, and equal volumes of methanol and acetonitrile were added. After vortexing, the protein was denatured and extracted. The mixture was then centrifuged at 14,400 rpm for 5 min. The supernatant was transferred to the EP tube, and the precipitate was dissolved in an equal volume of PBS buffer; then an equal volume of methanol and acetonitrile was added according to the above operation, and then vortexed and centrifuged. The supernatant was combined in the EP tube, and the precipitate was subjected to the same treatment (a total of 3 times). Finally, the fluorescence intensity of the combined supernatant was measured using a microplate reader. The concentration of the probe was obtained from the standard curve (simulated physiological environment). The content of the probe in each tissue was divided by the total injection volume, and then divided by the weight of each tissue to obtain the % ID/g value of the probe in each tissue. This value in the brain tissue can represent brain permeability, and the ratio of different time values can represent the brain clearance rate. The value of % ID/g of different tissues at different times can reflect the tissue distribution of the probe.

### 3.9. Determination of Binding Constant of Probe with Aβ Aggregates In Vitro

The saturation curve method was used to determine the binding constant K_d_ value. The probe was dissolved in DMSO to prepare a 10 mM DMSO stock solution, and then diluted with PBS to different concentrations for use. In a 96-well plate, 100 μL of probe diluent (final concentrations of 1000 nM, 500 nM, 250 nM, 50 nM, 25 nM, 10 nM) was added to each well, and 100 μL of prepared Aβ aggregates (final concentration of 3.0 μM) was added. The 96-well plate was shaken and incubated at 37 °C for 1 h. After incubation, fluorescence detection was performed using a full-band microplate reader (Tecan Austria GmbH, SPARK). The saturation titration curve and binding constant of each probe to Aβ aggregates were obtained by fitting the concentration gradient and fluorescence intensity of the probe by Prism 8.3.0. The data were analyzed by nonlinear regression using a one-site specific binding model (the “One site—Specific binding” equation in the “Binding—Saturation” family).

### 3.10. Binding Selectivity Test of Probe with Aβ Aggregates In Vitro

The probe was accurately weighed and dissolved in DMSO to prepare a 10 mM DMSO stock solution, which was diluted with PBS. Aβ_1–42_ (human sequence DAEFRHDSGYEVHHQKLVFFAEDVGSNKGAIIGLMVGGVVIA; ≥95% HPLC; Sigma-Aldrich, cat. A9810; calculated molecular weight 4514.04 Da; CAS 107761-42-2) was used throughout this study. Following the protein incubation protocol previously established in our group [42], Aβ_1–42_ was used as the Aβ species in this study. Protein samples were prepared as follows. For the preparation of Aβ_1–42_ monomer, Aβ_1–42_ (1 mg) was dissolved in HFIP (1 mL) and divided into 10 aliquots. HFIP was evaporated to dryness in a fume hood, and the aliquots were stored at −80 °C. Immediately before use, one aliquot was diluted to 50 μM with PBS (50 mM, pH 7.4). For the preparation of Aβ_1–42_ oligomer, Aβ_1–42_ (1 mg) was dissolved in HFIP (1 mL) and divided into 10 aliquots. HFIP was evaporated to dryness in a fume hood, and the aliquots were stored at −80 °C. One aliquot was diluted to 100 μM with PBS (50 mM, pH 7.4), left to stand at 4 °C for 24 h, and then incubated at 37 °C for 1 h before use. For the Preparation of Aβ_1–42_ aggregates, it was dissolved in dry DMSO to 5 mM and immediately diluted to 100 μM in PBS (final DMSO =2%, *v*/*v*), and incubated at 37 °C for 7 days. In a 96-well plate, 100 μL of probe diluent (final concentration of 1.0 μM) was added to each well, and 100 μL of different metal ions, amino acids, Aβ aggregates, oligomers, and monomers (final concentration of 10 μM) were added. The 96-well plates were shaken and incubated at 37 °C for 1 h. After incubation, fluorescence detection was performed using a full-band microplate reader (Tecan Austria GmbH, SPARK). The bar chart is obtained by processing the data with Prism 8.3.0.

### 3.11. Binding Competition Experiment with ThT

The probe was configured with different final concentrations (0, 0.5, 1, 2, 3, 4, 5 μM) and added to the ThT @ Aβ aggregate solution to record the fluorescence spectra of the probe and ThT.

### 3.12. Binding Ability to Aβ Aggregates In Vivo

Near-infrared animal in vivo imaging experiments were performed using the IVIS Lumina III animal imaging system (Caliper Life Sciences, Hopkinton, MA, USA), and data analysis was performed using Living image 4.4. Group allocation was determined by genotype (APP/PS1 transgenic vs. wild-type, confirmed before the start of the study); because genotype is an intrinsic property of each animal, randomization was not applicable to this comparison. No blinding was applied. The primary readout, fluorescence intensity, was quantified directly by the imaging software (Living Image 4.4) using identical acquisition settings and a fixed region of interest in every animal, i.e., an automated instrument measurement that does not depend on observer judgment. The risk of detection bias is therefore considered low. No animals were excluded from the analysis. All animals completed the study, and no deaths, adverse events, or technical failures (e.g., failed tail-vein injection, unusable imaging data) occurred during the observation period. The number of animals reported in the Results therefore equals the number included at the start of the study. The experimental method is as follows: First, the hairs of the brains of each mouse (male APP/PS1 transgenic AD model mice, 10 months old and male wild-type control mice of the same age in the same litter, n = 3 per group) were removed. The formula of each probe injection was 10% ethanol, 5% Tween 80 and 85% PBS (PH = 7.4). The probe was injected into the tail vein of mice (4.0 mg/Kg). The IVIS Lumina III animal imaging system was used to collect the fluorescence signals of the mouse brain before injection (background signal) and 5, 10, 15, 20, 30, 60, 120 and 180 min after injection of the probe. Using Living image 4.4, the region of interest (ROI) selection and quantitative analysis of the same size were performed at the same position in each mouse‘s brain to quantify the data, which is used for data analysis to evaluate the imaging results.

## 4. Conclusions

In summary, we developed P3, a hemicyanine-derived near-infrared fluorescence probe that enables wash-free imaging of amyloid-β (Aβ) aggregates in Alzheimer’s disease (AD). P3 exhibited high binding affinity toward Aβ aggregates, and it specifically labeled Aβ plaques in brain slices with negligible background, achieving a high signal-to-noise ratio of 80 ± 4 without any washing step. Unlike the reference dye ThT, which indiscriminately stained normal neuronal cells, P3 selectively distinguished Aβ aggregates. In vivo, P3 clearly differentiated APP/PS1 AD mice from wild-type mice with a 4.8-fold signal ratio, together with adequate brain uptake and rapid clearance. These favorable properties stem from the amphiphilic fine-tuning of log *P* (1.32 ± 0.02), which balances brain permeability, clearance, and nonspecific binding. Overall, this study successfully developed a probe that integrates adequate brain uptake and clearance, high affinity, rapid visualization, and low imaging background. Moreover, it provides valuable insights for the currently limited design of wash-free NIRF probes targeting Aβ aggregates.

## Figures and Tables

**Table 1:** Physicochemical Properties of P1, P2, P3 and X6.

Name	λex (nm) ^a^	λem (nm)-Free ^a^	λem (nm)-Binding ^b^	Fold Increase ^c^	Log *P*	QY_DCM_ (%)
P1	645	780	715	47.6 ± 1.4	0.68 ± 0.01	0.83 ± 0.08
P2	655	819	780	11.1 ± 0.5	0.71 ± 0.01	0.16 ± 0.06
P3	650	785	720	131.4 ± 7.0	1.32 ± 0.02	2.34 ± 0.10
X6	610	770	708	40.0 ± 0.6	0.77 ± 0.01	1.03

^a^ Measured in PBS. ^b^ after binding to Aβ aggregates. ^c^ Fold increase in fluorescence intensity upon binding with Aβ aggregates.

## Data Availability

The experimental data used to support the results of this study are available in the article and in the Supplementary Materials.

## Supplementary Materials

The following supporting information can be downloaded at https://www.mdpi.com/article/10.3390/molecules31203624/s1, Figure S1: ^1^H NMR of A-1; Figure S2: ^1^H NMR of A-2; Figure S3: ^1^H NMR of A-3; Figure S4: ^1^H NMR of B; Figure S5: ^1^H NMR of C-1; Figure S6: ^1^H NMR of C-2; Figure S7: ^1^H NMR of C-3; Figure S8: ^1^H NMR of D; Figure S9: ^1^H NMR of P1; Figure S10: ^13^C NMR of P1; Figure S11: ^1^H NMR of P2; Figure S12: ^13^C NMR of P2; Figure S13: ^1^H NMR of P3; Figure S14: ^13^C NMR of P3; Figure S15: HRMS of Compound P1; Figure S16: HRMS of Compound P2; Figure S17: HRMS of Compound P3; Figure S18: Fluorescence spectra of probe P1 and P2 polarity sensitivity test; Figure S19: The fluorescence intensity of probe P1 and P2 at different time (0 min, 10 min, 30 min, 60 min, 120 min, 240 min and 360 min); Figure S20: The absolute fluorescence quantum yield of the probe; Figure S21: The standard curve of the probe; Figure S22: The fluorescence intensity of 1 μM Probe binding to 10 μM Aβ aggregates increased multiples in vitro. Error bars: mean ± standard deviation (SD) (*n* = 3); Figure S23: The fluorescence spectra of 1 μM probe in different concentrations of Aβ aggregates (0,2,4,6,8,10 μM) and the linear regression analysis of the maximum fluorescence intensity of the probe in different concentrations of Aβ aggregates; Figure S24: The binding affinity of 3 μM Aβ aggregates with 1000, 500, 250, 50, 25, 10 nM Probe in vitro; Figure S25: The binding selectivity of 1 μM Probe to 10 μM different metal ions, amino acids, Aβ aggregates, oligomers and monomers in vitro; Figure S26: The standard curve of the fluorescence intensity of probes P3 and X6 in the physiological environment of brain tissue with the change of probe concentration; Figure S27: The standard curve of P3 in different physiological environment (heart, liver, spleen, lung, kidney, brain); Table S1: Evaluation of the properties of hemicyanine probes targeting Aβ aggregates; Table S2: Prediction of the basic properties of probes; Table S3: Summary of the properties of wash-free probes.
