# Supplementary material for: Hemicyanine-Based Wash-Free Near-Infrared Fluorescent Probe for Imaging Amyloid-β Aggregates in Alzheimer’s Disease
Source: Molecules. 2026 Oct 11;31(20):3624. doi: 10.3390/molecules31203624 (PMC13707792; doi:10.3390/molecules31203624)
Supplement: Supplementary file 1 [file molecules-31-03624-s001.zip › molecules-4564991-supplementary.pdf]

Supporting information

Hemicyanine-Based Wash-Free Near-Infrared Fluorescent Probe for Imaging Amyloid-β Aggregates in Alzheimer's Disease

Table of contents:

1. **Evaluation of the properties of hemicyanine probes targeting Aβ aggregates.**
2. **NMR and HRMS spectra**
3. **Optical Properties**
4. **Log *P* test**
5. **Binding ability to Aβ aggregates *in vitro***
6. **Tissue distribution test**
7. **Prediction of the basic properties of probes**
8. **Summary of the properties of wash-free probes**

1. Evaluation of the properties of hemicyanine probes targeting Aβ aggregates.

Table S1. Evaluation of the properties of hemicyanine probes targeting Aβ aggregates.

Name	λex (nm)	λem (nm)	Mol.Wt	Fold Increase (*in vitro*)	Log *P*	Fold Increase (*in vivo*)
MC-1	630	695	375.90	28	3.70	1.16
ZT-1	625	752	449.04	18	-	1.50
MZ-2	610	725	400.55	15.35	1.37	1.23
NS	601	780	481.72	18.7	0.92	1.98
X6	610	770	555.11	42.36	0.769	3.79

2. NMR and HRMS spectra

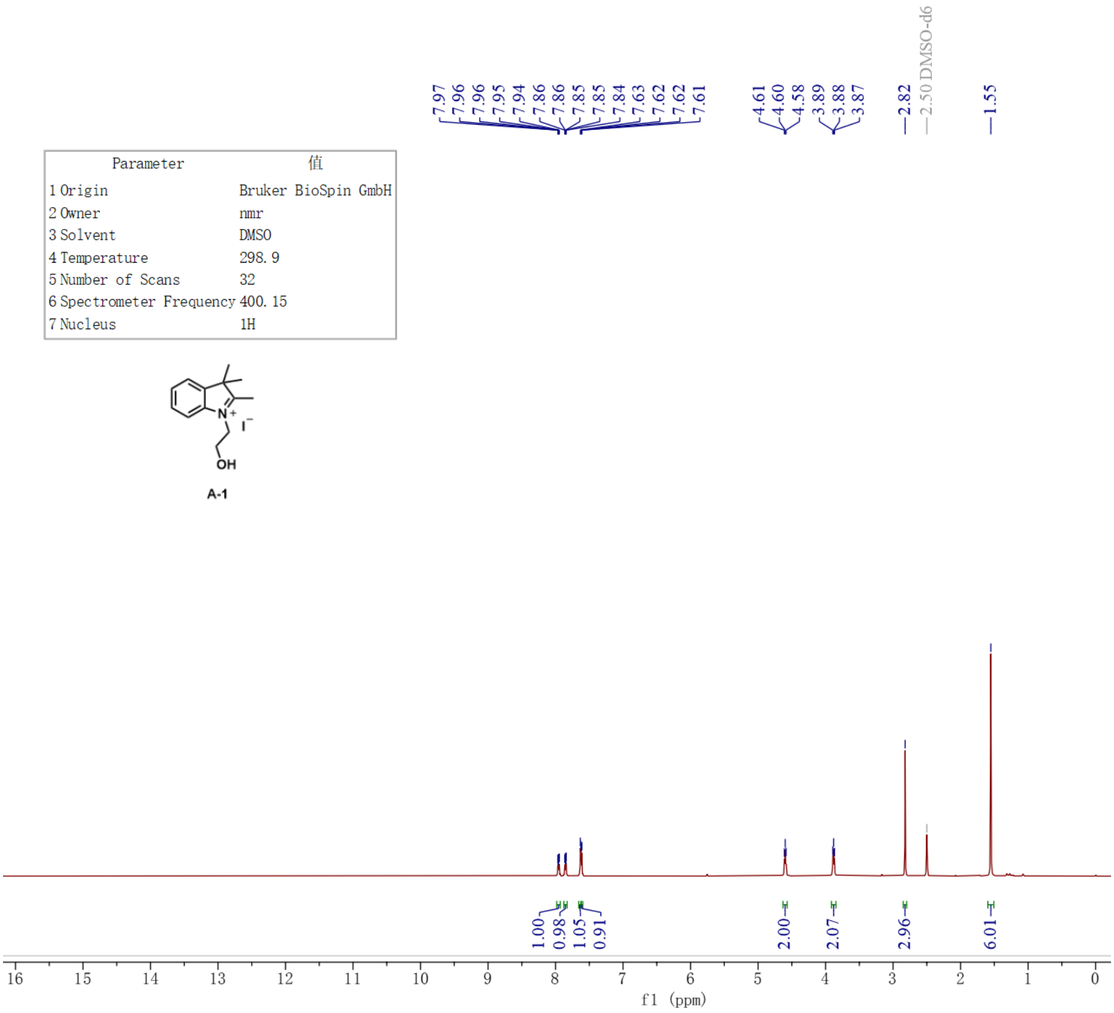

Figure S1 ^{1}H NMR of A-1.

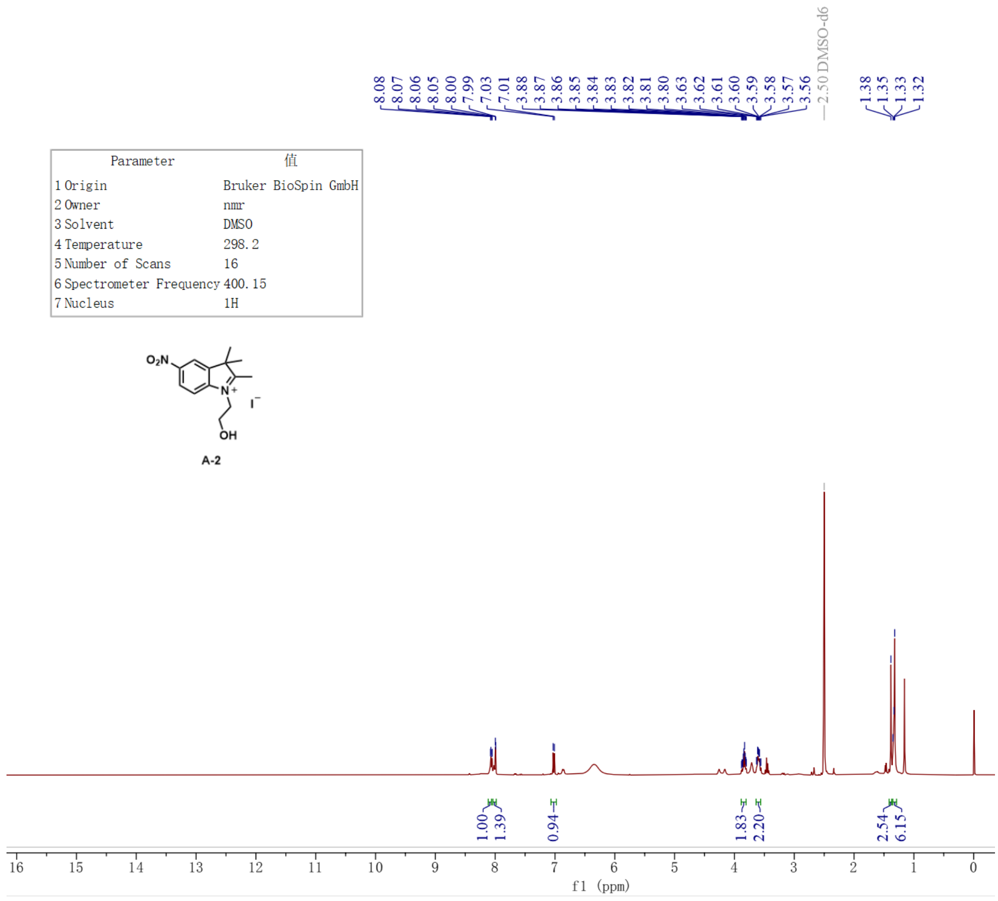

Figure S2 ^{1}H NMR of A-2.

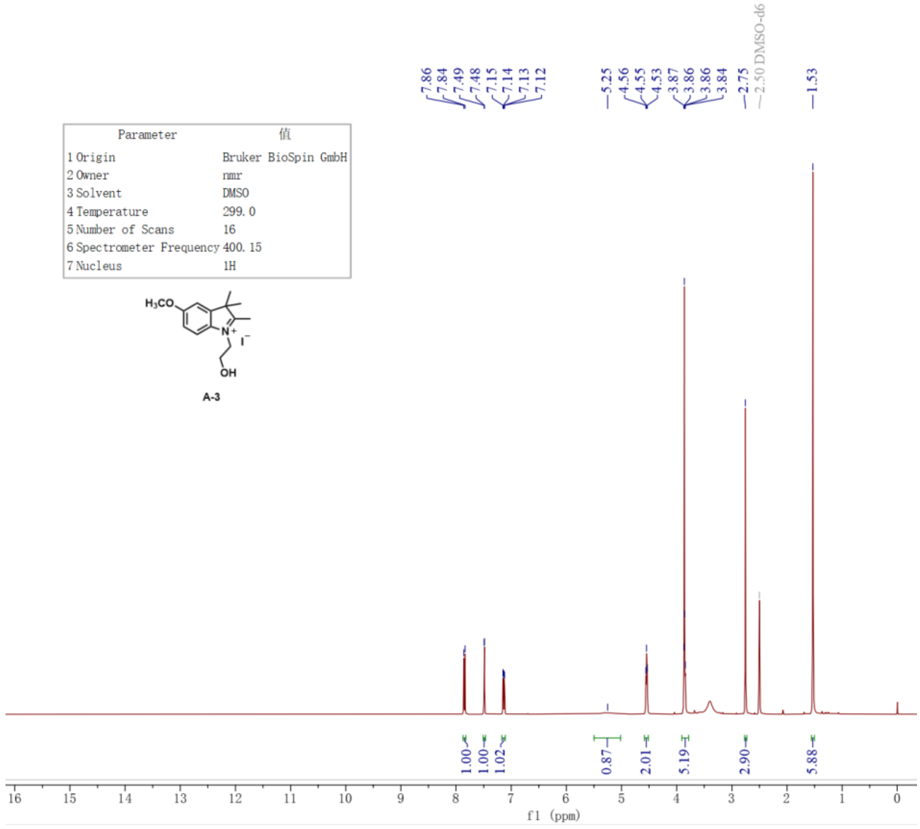

Figure S3 ^{1}H NMR of A-3.

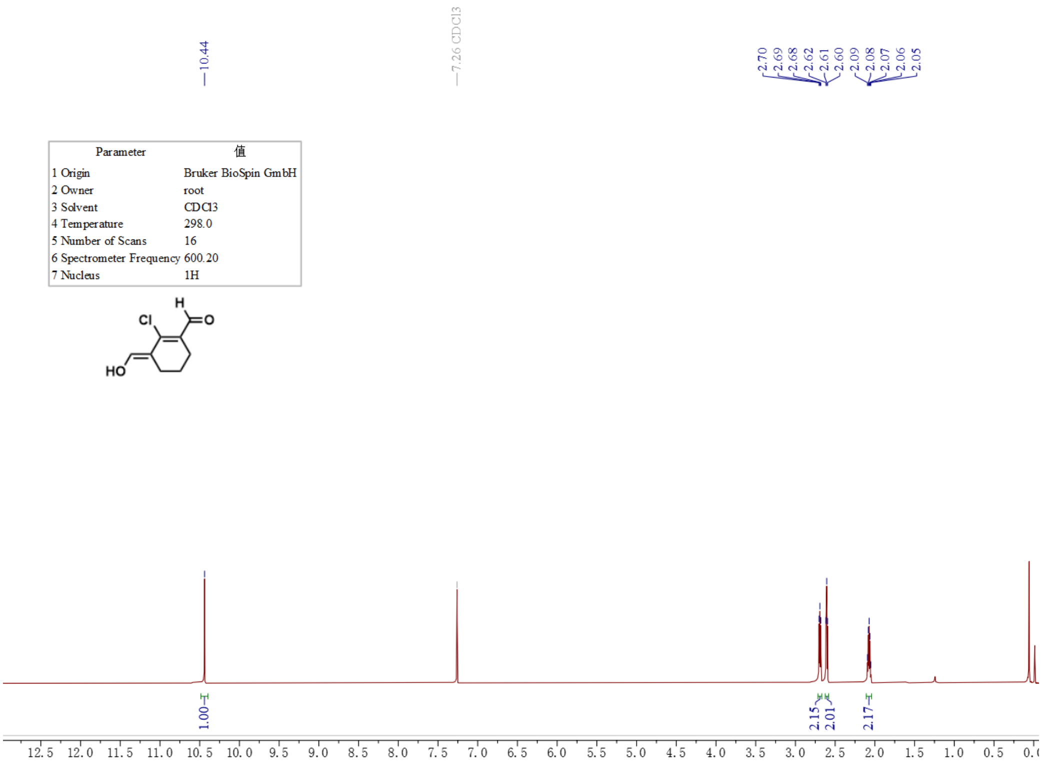

Figure S4 ^{1}H NMR of B.

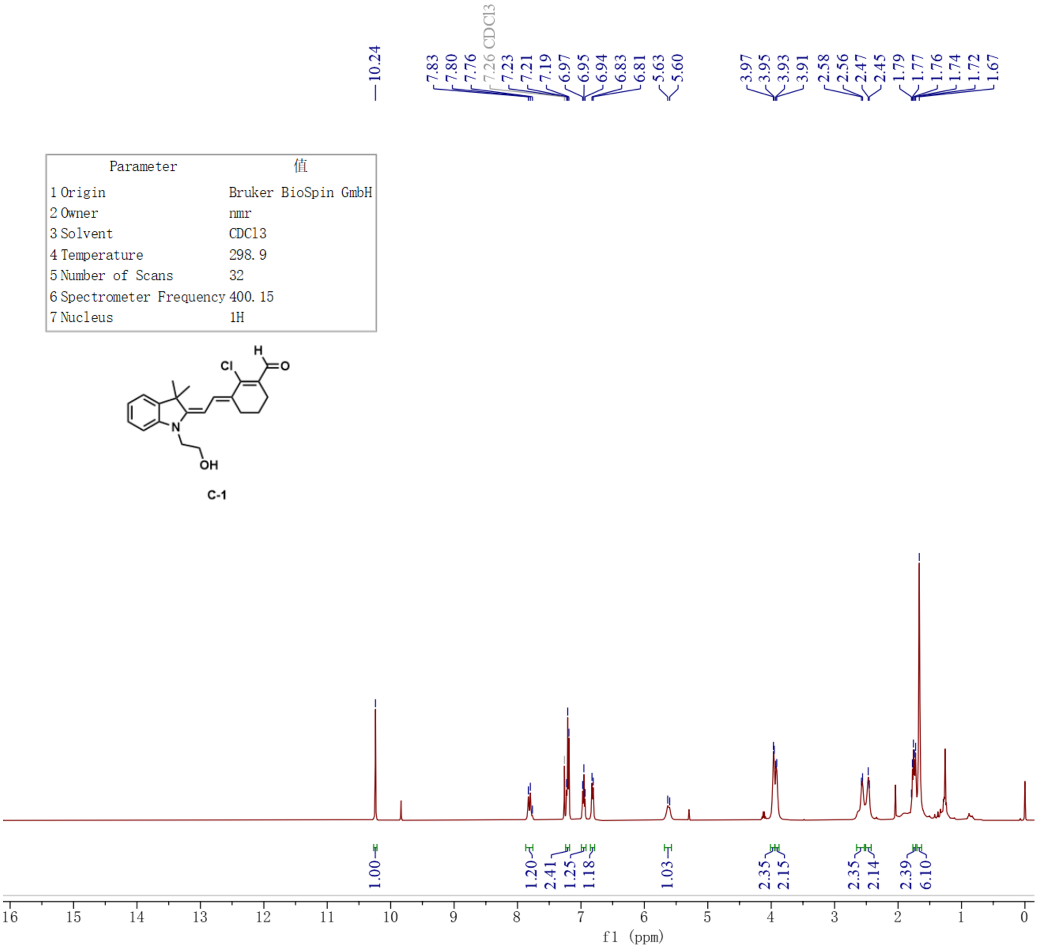

Figure S5 ^{1}H NMR of C-1.

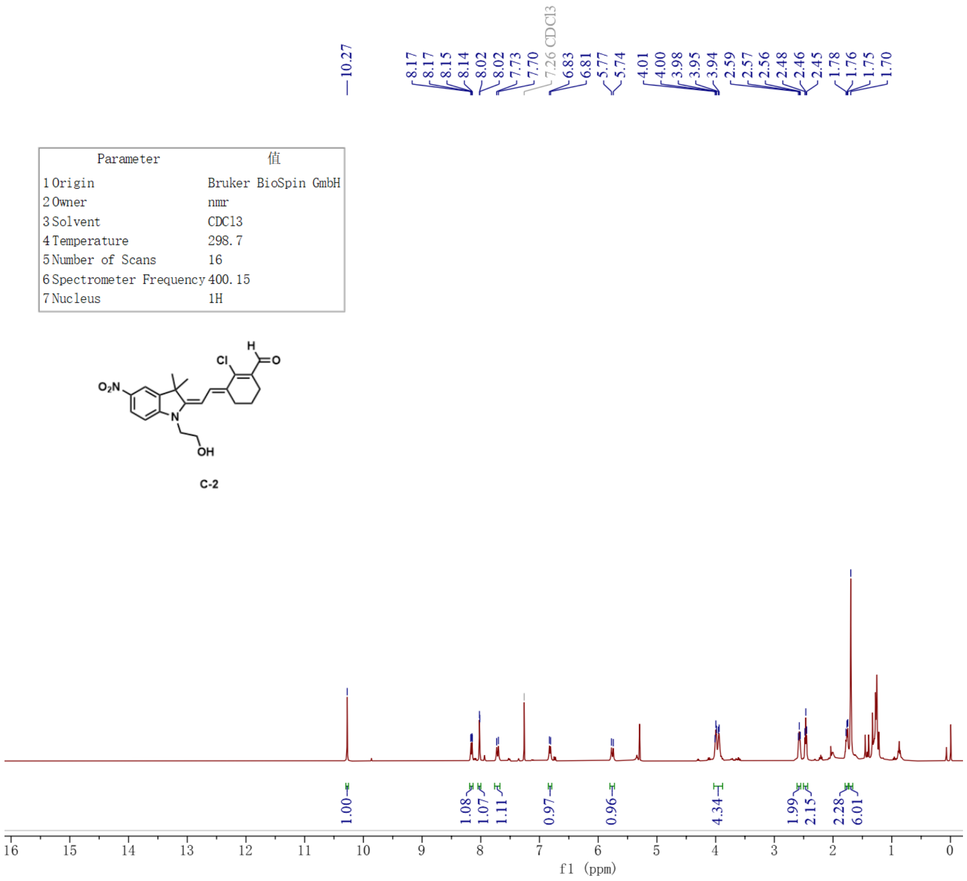

Figure S6 ^{1}H NMR of C-2.

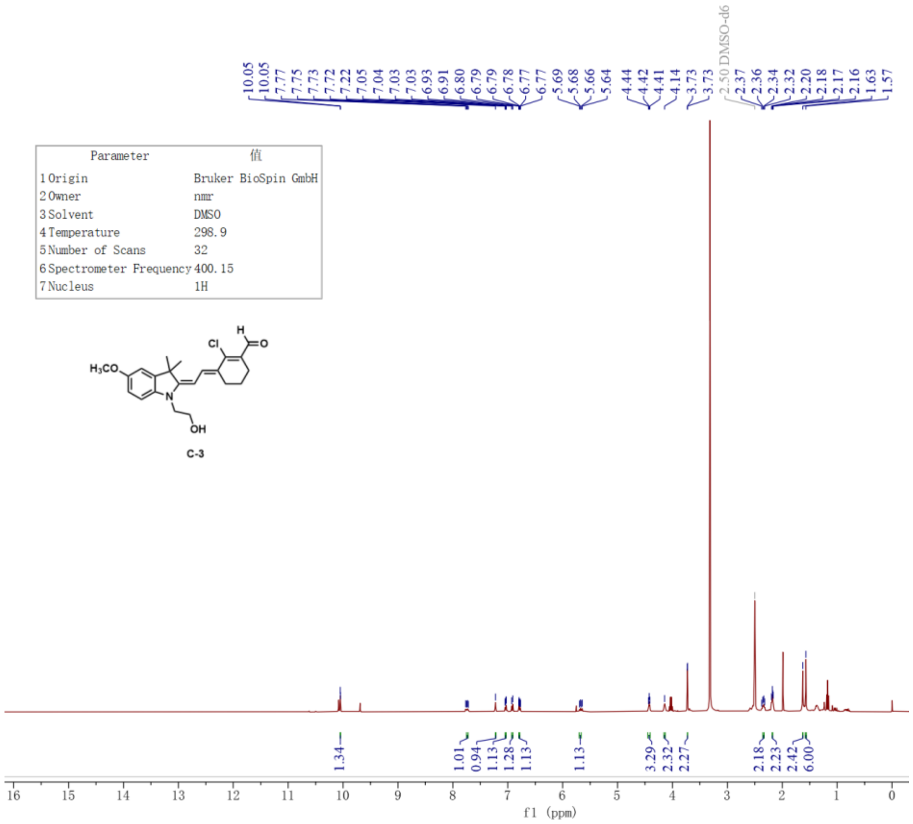

Figure S7 ^{1}H NMR of C-3.

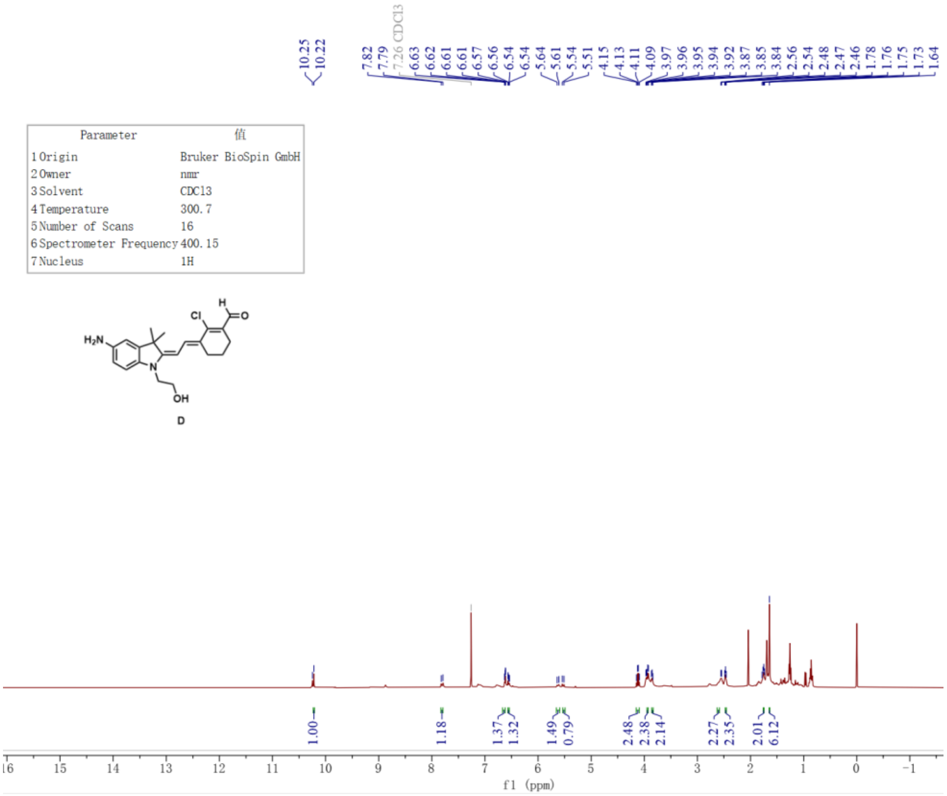

Figure S8 ^{1}H NMR of D.

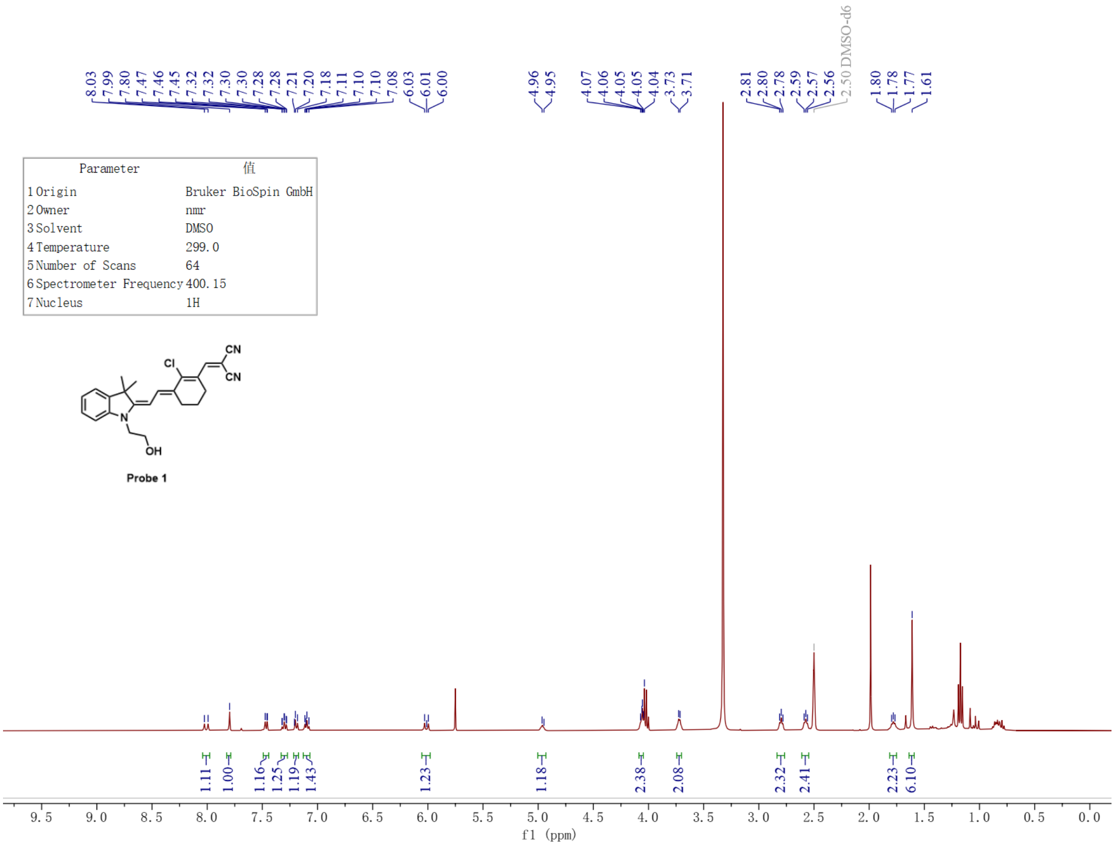

Figure S9 ^{1}H NMR of P1.

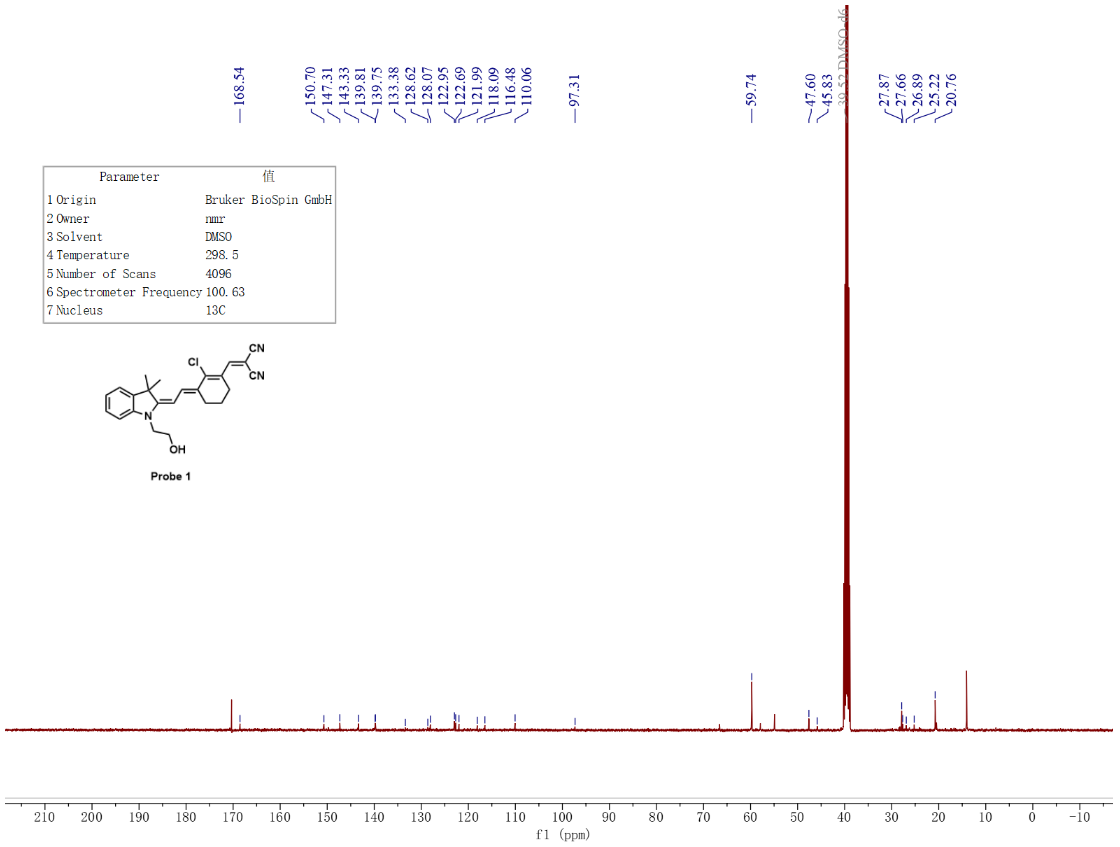

Figure S10 ^{13}C NMR of P1.

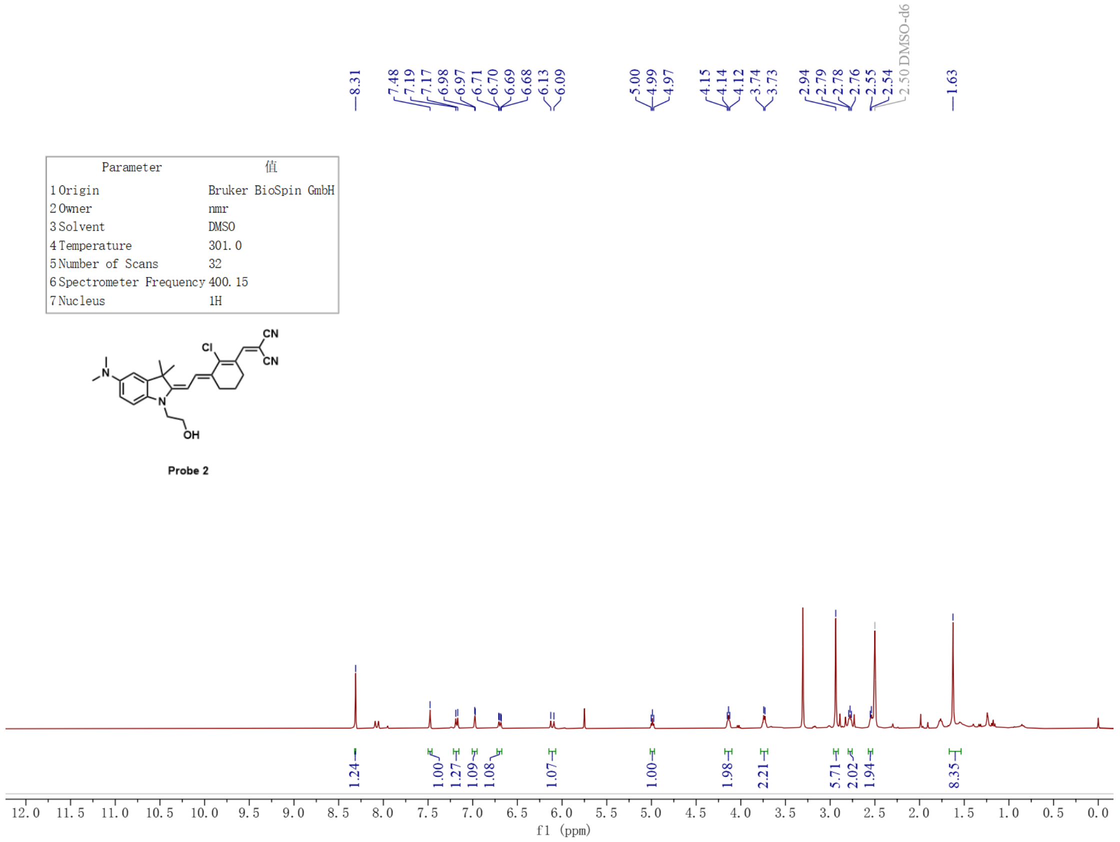

Figure S11 ^{1}H NMR of P2.

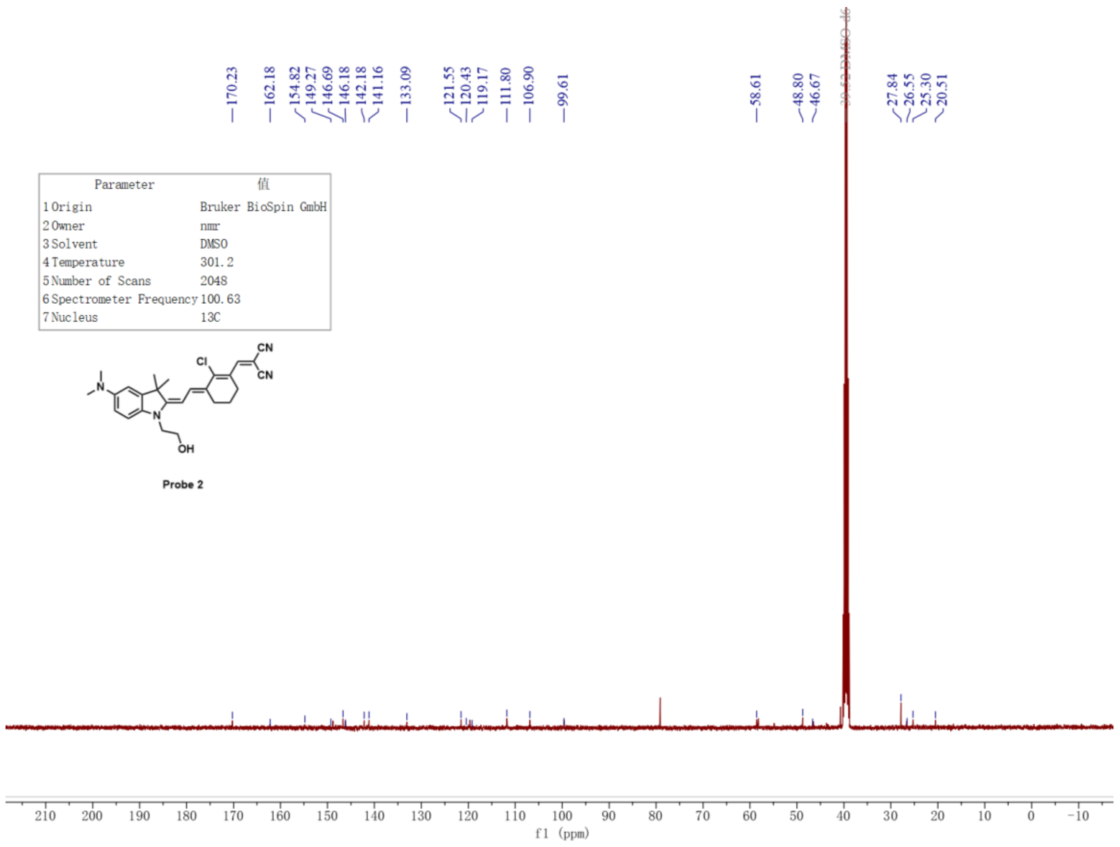

Figure S12 ^{13}C NMR of P2.

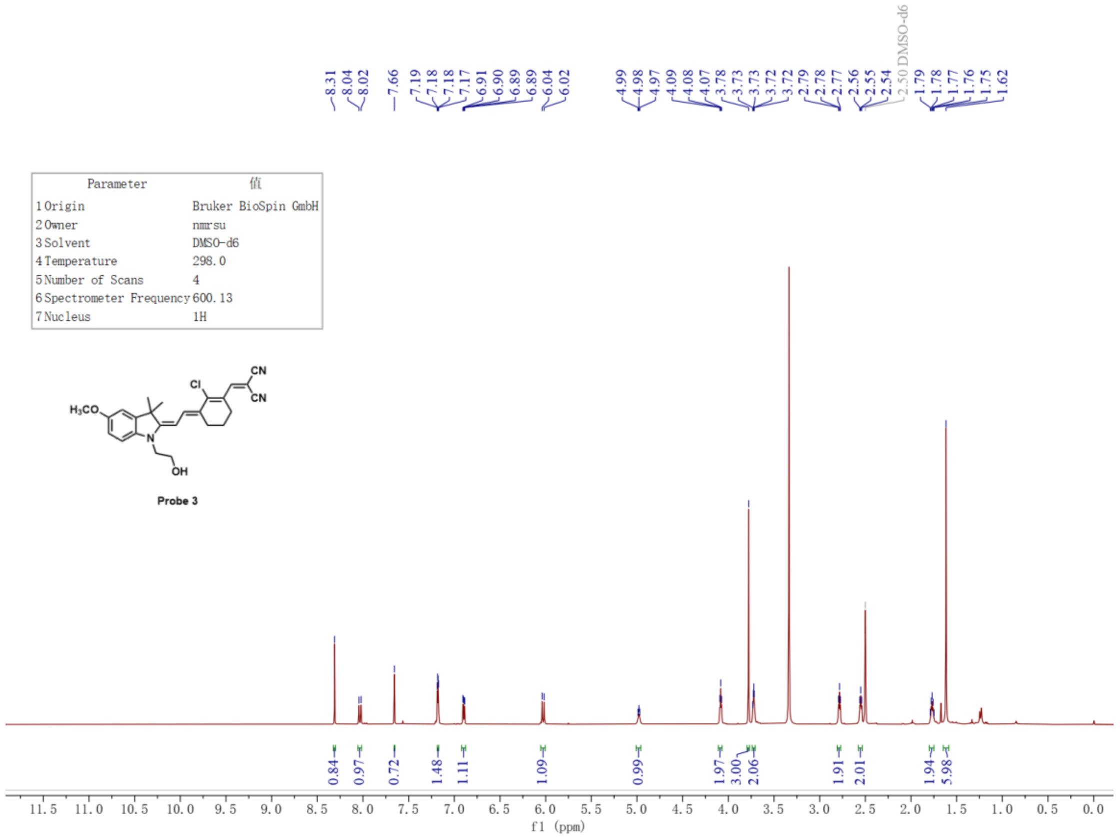

Figure S13 ^{1}H NMR of P3.

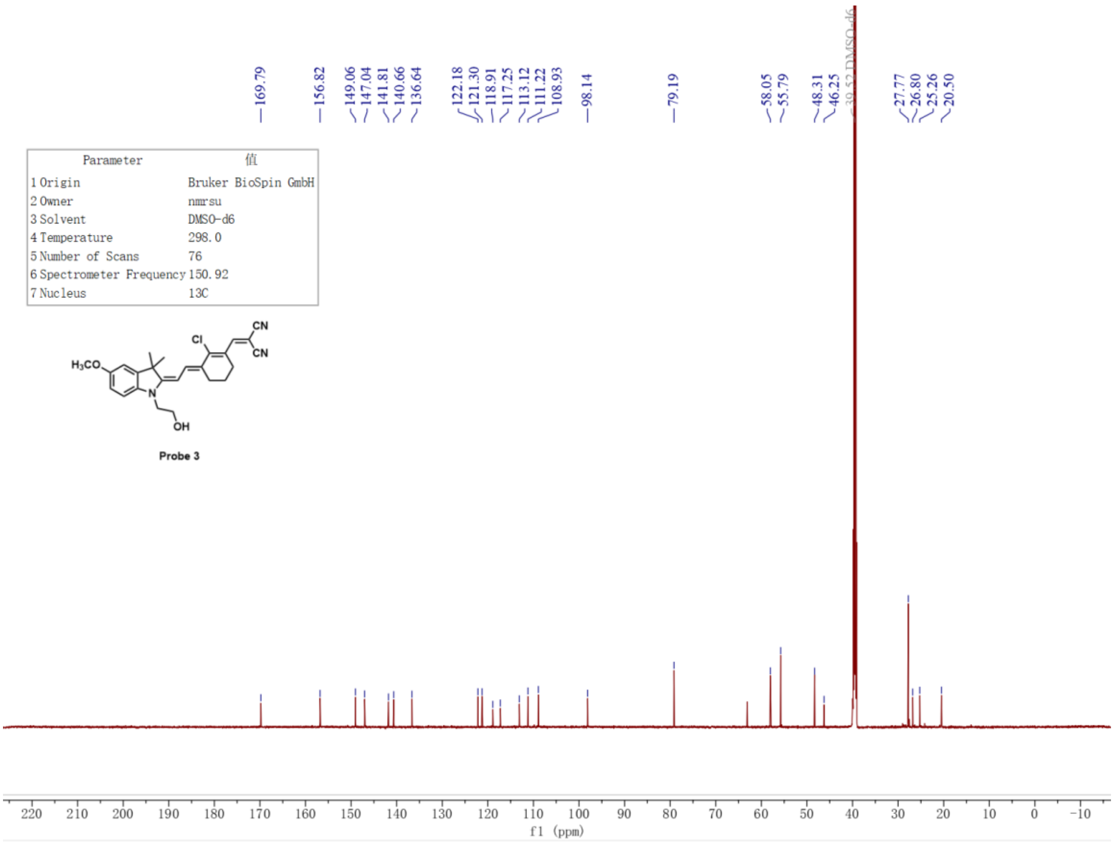

Figure S14 ^{13}C NMR of P3.

Elemental Composition Report **Page 1**

Single Mass Analysis
Tolerance = 5.0 mDa / DBE: min = -1.5, max = 50.0
Element prediction: Off
Number of isotope peaks used for i-FIT = 3

Monoisotopic Mass, Even Electron Ions
311 formula(e) evaluated with 1 results within limits (up to 50 best isotopic matches for each mass)
Elements Used:
C: 24-24 H: 25-25 N: 0-100 O: 0-100 Cl: 1-1
260511-3-P1 9 (0.072)
1: TOF MS ES+

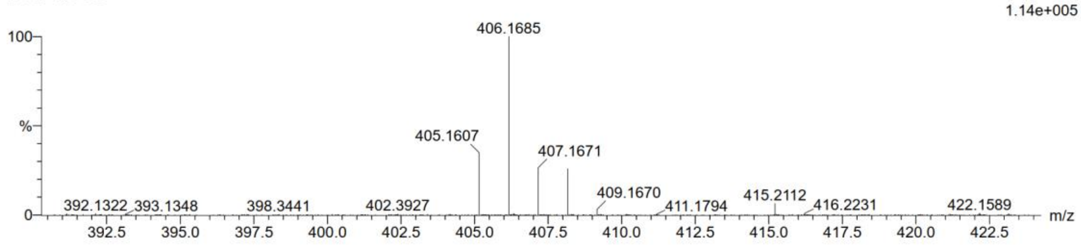

Minimum:				-1.5					
Maximum:		5.0	10.0	50.0					
Mass	Calc. Mass	mDa	PPM	DBE	i-FIT	Norm	Conf(%)	Formula	
406.1685	406.1686	-0.1	-0.2	13.5	455.3	n/a	n/a	C24 H25 N3 O Cl	

Figure S15 HRMS of Compound P1.

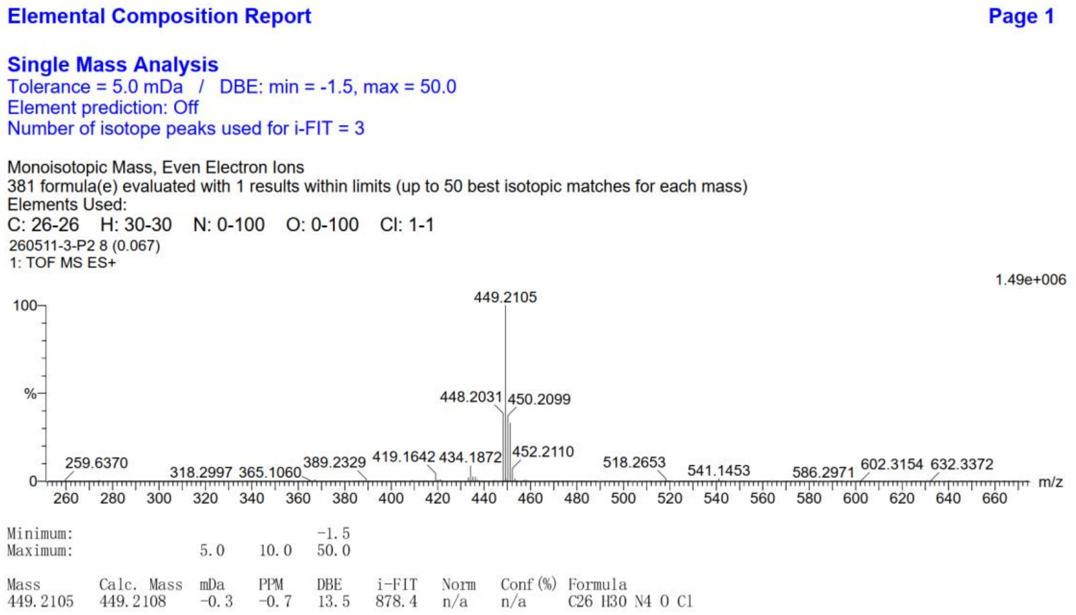

Figure S16 HRMS of Compound P2.

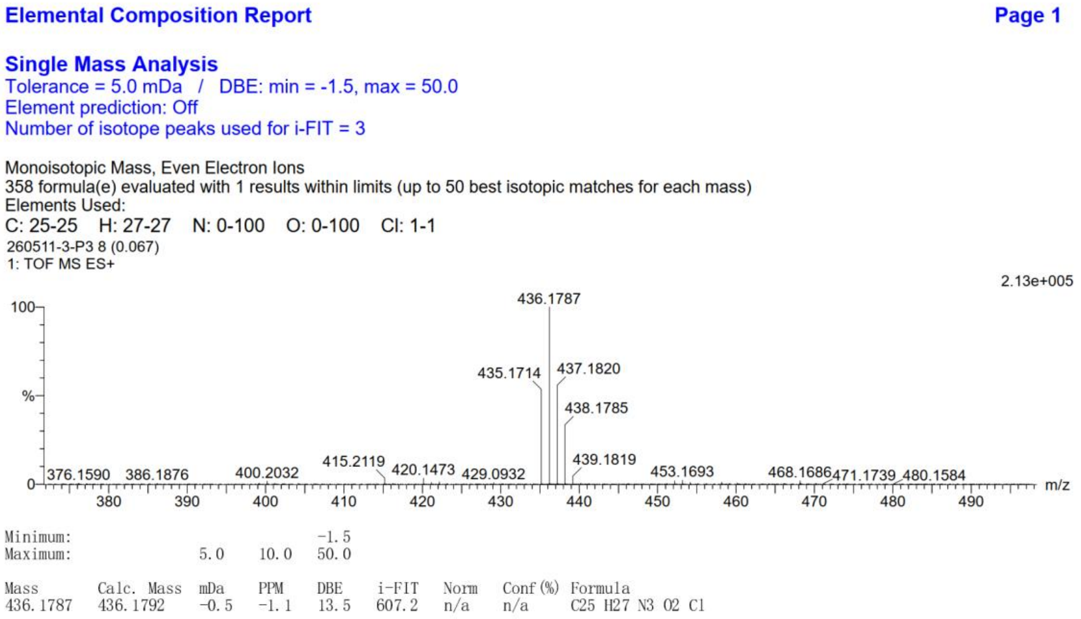

Figure S17 HRMS of Compound P3.

3. Optical Properties

3.1 Polarity sensitivity test

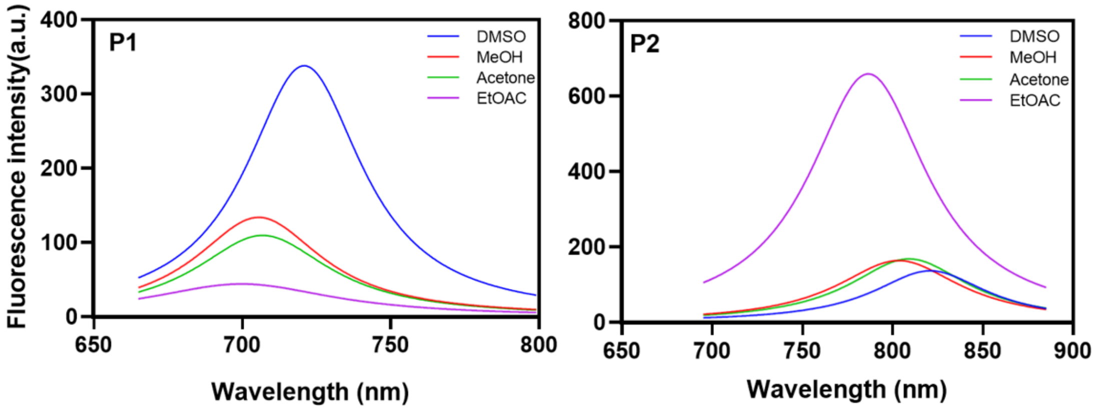

Figure S18 Fluorescence spectra of probe P1 and P2 polarity sensitivity test.

3.2 Light Stability

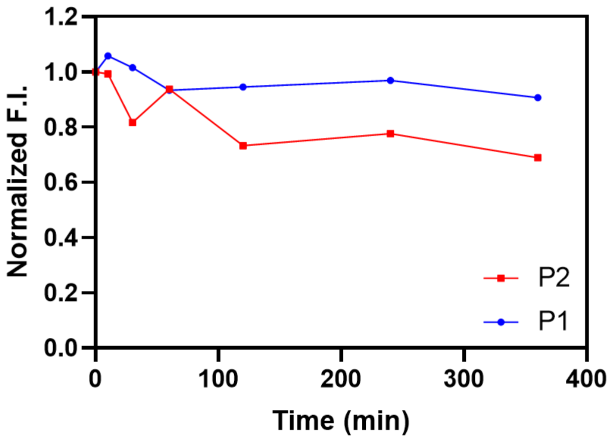

Figure S19 The fluorescence intensity of probe P1 and P2 at different time (0 min, 10 min, 30 min, 60 min, 120 min, 240 min and 360 min).

3.3 Absolute fluorescence quantum yield test

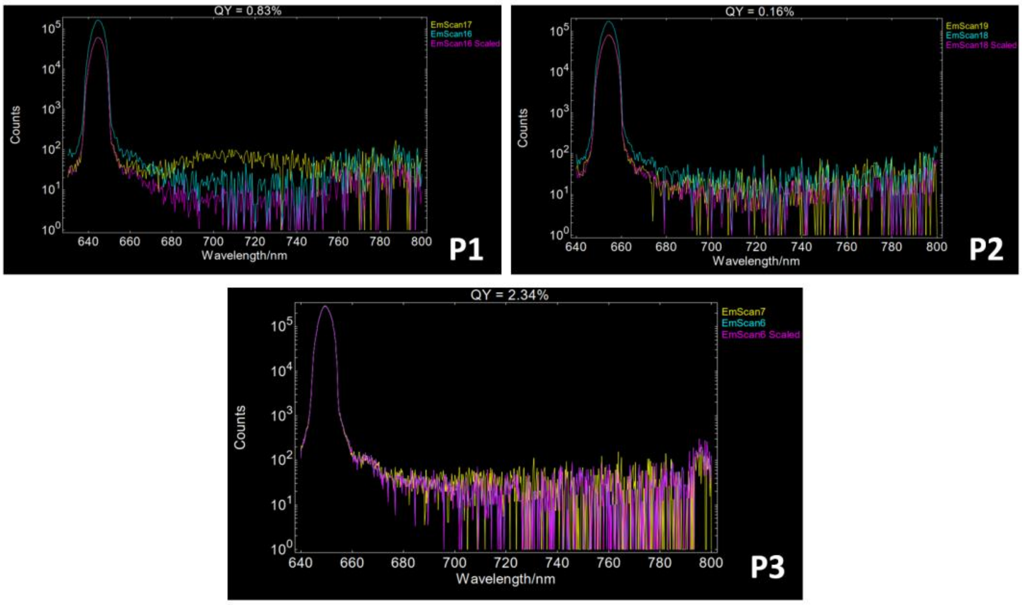

Figure S20 The absolute fluorescence quantum yield of the probe.

4. Log *P* test

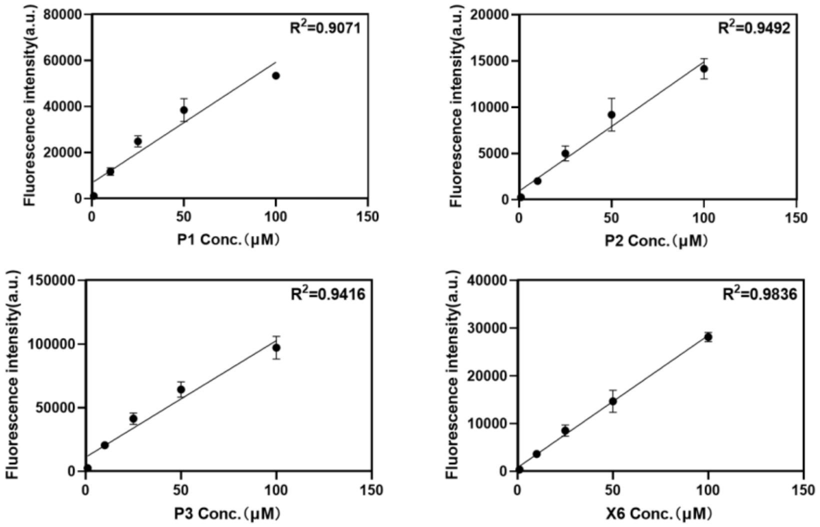

Figure S21 The standard curve of the probe.

5. Binding ability to Aβ aggregates *in vitro*

5.1 Fluorescence spectra of the probe after binding to Aβ aggregates

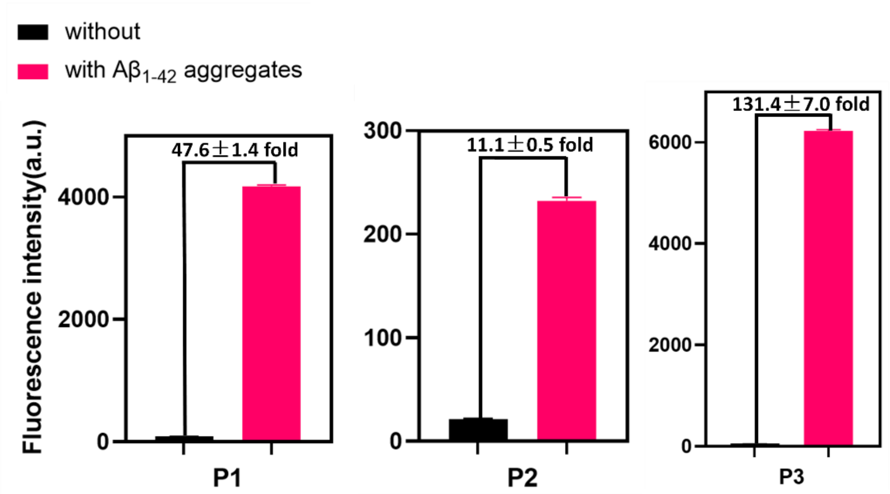

Figure S22 The fluorescence intensity of 1 μM Probe binding to 10 μM Aβ aggregates increased multiples *in vitro*. Error bars: mean ±standard deviation (SD) (n = 3).

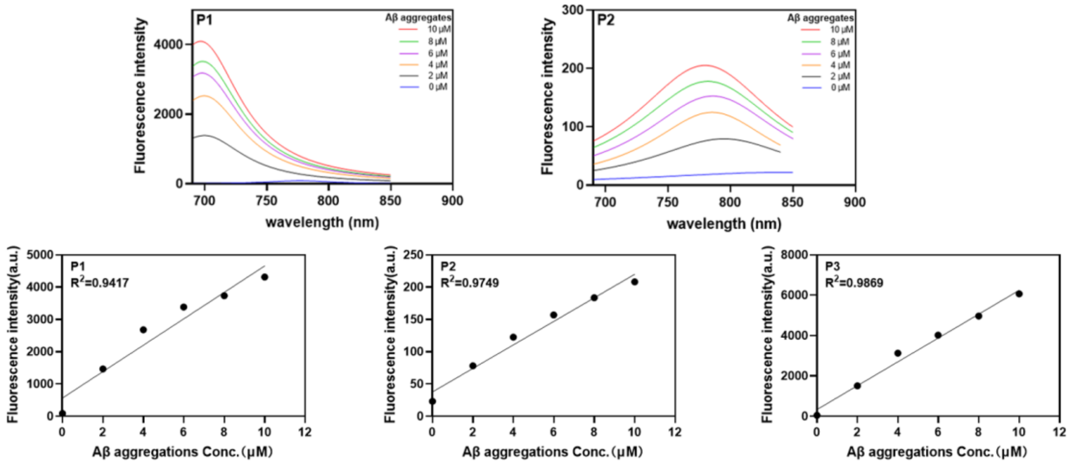

Figure S23 The fluorescence spectra of 1 μM probe in different concentrations of Aβ aggregates (0,2,4,6,8,10 μM) and the linear regression analysis of the maximum fluorescence intensity of the probe in different concentrations of Aβ aggregates.

5.2 Determination of binding constant (K_d test)

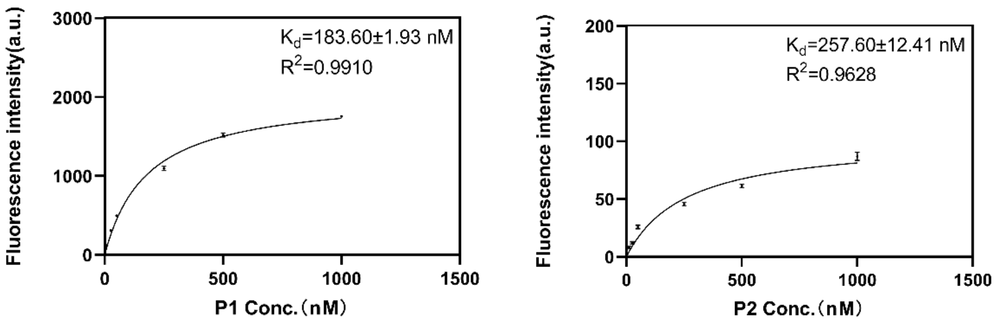

Figure S24 The binding affinity of 3μM Aβaggregates with 1000, 500, 250, 50, 25, 10nM Probe *in vitro*.

5.3 Binding selectivity test

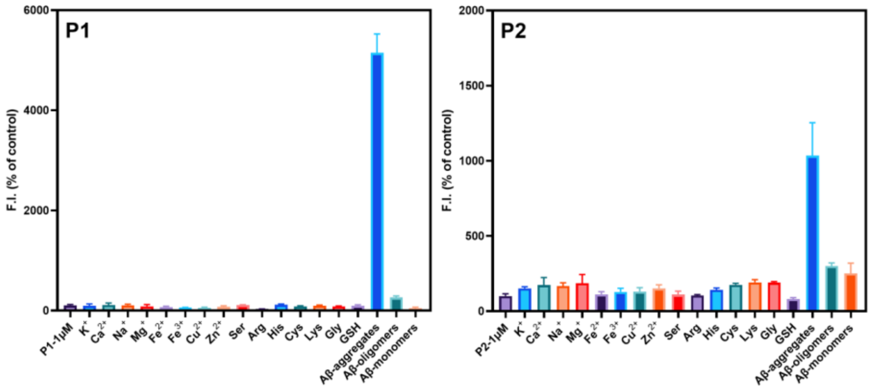

Figure S25 The binding selectivity of 1 μM Probe to 10 μM different metal ions, amino acids, Aβ aggregates, oligomers and monomers *in vitro*.

6. Tissue distribution test

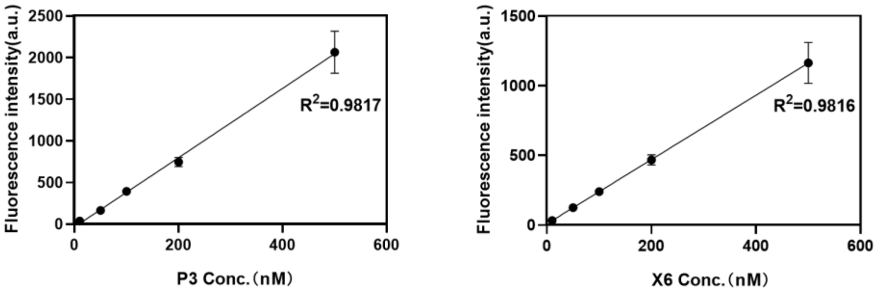

Figure S26 The standard curve of the fluorescence intensity of probes P3 and X6 in the physiological environment of brain tissue with the change of probe concentration.

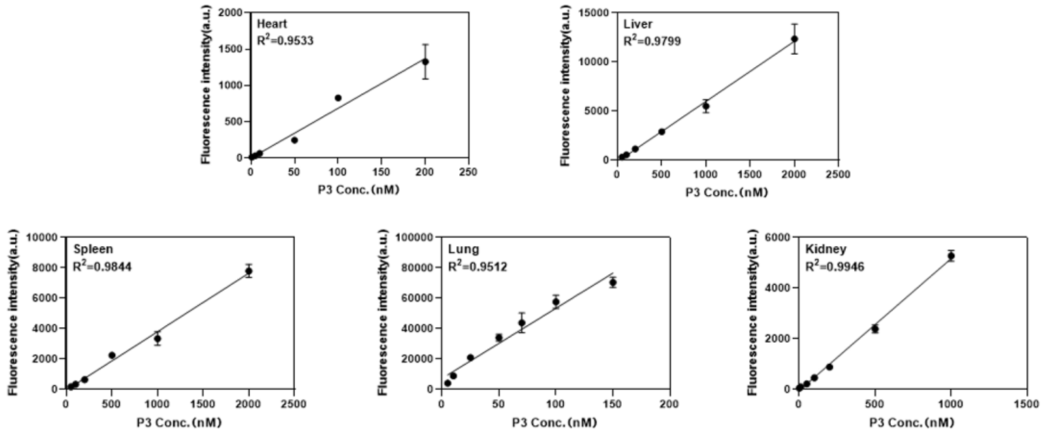

Figure S27 The standard curve of P3 in different physiological environment (heart, liver, spleen, lung, kidney, brain).

7. Prediction of the basic properties of probes

Table S2. Prediction of the basic properties of probes

	pKa	Log *S* (ESOL)	Solubility
P1	13.822	-5.06	3.57e-03 mg/ml ; 8.80e-06 mol/l
P2	13.852	-5.32	2.13e-03 mg/ml ; 4.74e-06 mol/l
P3	13.797	-5.15	3.06e-03 mg/ml ; 7.03e-06 mol/l

8. Summary of the properties of wash-free probes

Table S3. Summary of the properties of wash-free probes

	scaffold	emission wavelength (nm)	fold enhancement (in vitro)	Log *P*= log(Co/Cw)	SNR (wash-free)	mouse model	fold enhancement (in vivo)
PZ-PYC6	styrene pyridine	603	7.6	-0.17±0.02	58.61 ± 4.35	-	-
CT-PYC6	styrene pyridine	595	27.1	-0.19±0.01	51.88 ± 2.91	-	-
DM-V2CN-PYC3	π-bridge skeleton of butadiene	696	30.0	-0.20±0.01	48.72	9-month-old 5xFAD	-
P3	Hemicyanine	785	131.4±7.0	1.32±0.02	80 ± 4	10-month-old APP/PS1	4.8
